# Proteome sequence features carry signatures of the environmental niche of prokaryotes

**DOI:** 10.1186/1471-2148-11-26

**Published:** 2011-01-26

**Authors:** Zlatko Smole, Nela Nikolic, Fran Supek, Tomislav Šmuc, Ivo F Sbalzarini, Anita Krisko

**Affiliations:** 1 Institute for Cell Biology, ETH Zuerich, Schafmattstrase 18, 8093 Zuerich, Switzerland; 2 Mediterranean Institute for Life Sciences, Mestrovicevo setaliste bb, 21000 Split, Croatia; 3 Institute of Biogeochemistry and Pollutant Dynamics, ETH Zuerich, Unversitätstrasse 16, 8092 Zuerich, Switzerland; 4 Division of Electronics, Rudjer Boskovic Institute, Bijenicka 54, 10000 Zagreb, Croatia; 5 Institute of Theoretical Computer Science and Swiss Institute of Bioinformatics, ETH Zurich, Zurich, Switzerland; 6 Institut National de la Santé et de la Recherche Médicale U1001, Université Paris Descartes, Faculté de Médecine, 156 rue de Vaugirard, 75730 Paris Cedex 15, France

## Abstract

**Background:**

Prokaryotic environmental adaptations occur at different levels within cells to ensure the preservation of genome integrity, proper protein folding and function as well as membrane fluidity. Although specific composition and structure of cellular components suitable for the variety of extreme conditions has already been postulated, a systematic study describing such adaptations has not yet been performed. We therefore explored whether the environmental niche of a prokaryote could be deduced from the sequence of its proteome. Finally, we aimed at finding the precise differences between proteome sequences of prokaryotes from different environments.

**Results:**

We analyzed the proteomes of 192 prokaryotes from different habitats. We collected detailed information about the optimal growth conditions of each microorganism. Furthermore, we selected 42 physico-chemical properties of amino acids and computed their values for each proteome. Further, on the same set of features we applied two fundamentally different machine learning methods, Support Vector Machines and Random Forests, to successfully classify between bacteria and archaea, halophiles and non-halophiles, as well as mesophiles, thermophiles and mesothermophiles. Finally, we performed feature selection by using Random Forests.

**Conclusions:**

To our knowledge, this is the first time that three different classification cases (domain of life, halophilicity and thermophilicity) of proteome adaptation are successfully performed with the same set of 42 features. The characteristic features of a specific adaptation constitute a signature that may help understanding the mechanisms of adaptation to extreme environments.

## Background

The availability of complete proteome sequences allows an in-depth comparison of their singleresidue compositions. Over 1000 proteomes of bacteria and archaea have recently become available, as they were derived from the respective genome sequences. Analysis of proteomes has already proven useful in prediction of structure and function of proteins as well as phylogenetic analysis. Moreover, it is the availability of complete proteome data that fuels the success of complementary per-proteome approaches to address global properties of microorganisms. Since amino-acid composition is principally determined by the structural and functional requirements of a given protein, one might expect it to correlate with a variety of factors. Conditions such as temperature, salt concentration, pH, and pressure within an organism's environmental niche are surely among the most important factors that cause selective pressure on the proteins evolving in different niches. Not all conditions are equally acceptable to all species: life is possible from -15°C to 113°C [1,2], up to 5.1 M NaCl [3], pH from 0 to 13 [4], etc. In this context, extreme conditions of life are those that exceed conditions for growth and reproduction that are optimal for the majority of organisms. Organisms that thrive in or even require extreme conditions are termed extremophiles. There are many different classes of extremophiles, corresponding to the way the environmental niche differs from that of the majority of mesophile organisms [5]. These classifications are not mutually exclusive, thus many extremophiles fall into multiple categories. Regardless of the environmental niche, however, adaptation and maintenance of protein integrity and function seems to be fundamental to survival of entire organisms [6-11]. Therefore, the physico-chemical properties of individual amino-acids, as well as whole proteins and proteomes, from various environmental niches should be explored in more detail.

In the past decade, many *in silico *studies on proteomes have mainly focused on functional annotation of individual proteins. The global characterization of a specie's lifestyle has received far less attention, with studies mostly focusing on gene content analysis, GC content, synteny analysis, but rarely on monitoring proteomic features. Moreover, previously reported studies usually relied on a small set of proteomes, which does not support comprehensive statistical analysis. A small number of proteomic features was usually analyzed without thorough quantification of feature relevance. Such studies have suggested that amino acid composition is one of the most important determinants of adaptations to extreme temperatures and high salt concentration.

Here, we use extensive machine learning studies in order to investigate whether and how the environmental niche of a prokaryote is reflected in the sequence of its proteome. The properties of each proteome were described in as much detail as possible by 42 proteomic features based on physico-chemical properties of individual amino acids. Based solely on proteome sequences, we were able to distinguish between bacteria and archaea as well as to describe the adaptation of bacteria and archaea to various conditions of the environment: from normal to high temperature (mesophiles vs. mesothermophiles vs. thermophiles) and from normal to high salt concentration (non-halophiles vs. halophiles). In order to detect biologically meaningful patterns in proteomes, we used two conceptually different supervised machine learning algorithms for data classification: Support Vector Machines (SVM) [12] and Random Forests (RF) [13]. The use of supervised techniques, as opposed to previous uses of unsupervised learning (clustering, principal component analysis, factor analysis) allowed us to greatly extend the set of used proteomes descriptors. Among them, the most relevant features have been detected and their importance quantified in order to gain insight into the structural and functional adaptation of proteins to the environment.

This large-scale analysis of the available proteomes of bacteria and archaea helps gaining a global understanding of the adaptation of proteomes to different environmental conditions.

## Results

### Classification accuracies varied from very good to excellent

The dataset (Additional file 1) used in this study consisted of:

1107 prokaryotic species divided and tagged according to their domain of life - archaea (82) and bacteria (1025);

192 prokaryotic species divided and tagged according to their optimal growth temperature range (thermophilicity) - mesophiles (142), mesothermophiles (22), thermophiles (23), unknown (5)

192 prokaryotic species divided and tagged according to their optimal growth NaCl concentration range (halophilicity) - non-halophiles (129) and halophiles (63)

We have applied Random Forests (RF) and Support Vector Machines (SVM) to each of these classification cases. The supervised classification algorithms learned the environmental signatures in the proteome features on the training data (2/3 of all data) and were then tested on the remaining third of the data with the class labels removed. This was repeated ten times with different randomly selected training and tests sets. The classification quality was measured on the test sets by counting the true positive rate (number of times the classifier could correctly predict the class label from the feature signatures learned on the training data) and the false positive rate (number of times a false alarm was given). The results are shown as Receiver Operating Characteristic (ROC) graphs (Additional File 2) with the associated Area Under the Curve (AUC) (Table 1). Perfect classification would lead to an AUC value of 1, indicating a 100% hit rate with no false alarms. This would mean that the lifestyle of the organisms could be perfectly predicted from its proteome features. AUC values between 0.8 and 0.9 are commonly considered very good, and values between 0.9 and 1.0 excellent [14]. A value of 0.5 corresponds to pure random guessing.

**Table 1:** Classification accuracies displayed as area under the curve (AUC) obtained by the support vector machines (SVM) and random forrest (RF) for the classification according to domain of life, halophilicity and thermophilicity.

	Area Under the Curve (AUC)	
	**SVM**	**RF**

Domain of Life	0.99	0.99

Halophilicity	0.83	0.89

Thermophilicity	0.96	0.95

In our experiments, the classification performance with respect to the domain of life and the optimal growth temperature range was excellent. The halophilicity of an organism was predicted "very good" from the proteome features by both classification methods. For the classification according to thermophilicity we have constructed 3 ROC curves using the one-against-one approach to the three-class problem (Additional File 3). The average AUCs are listed in Table 1.

Classification accuracies were excellent for both methods we applied. Classification using multiclass SVM was less good (data not shown).

The results obtained from SVM and RF do not significantly differ. This suggests that they are independent of the classification method and reflect signatures in the data rather than artifacts of the classification algorithms.

As a control for each classification case, we randomly permuted the values in the input vectors. The AUC then dropped to approximately 0.5, indicating that there is no relationship between the predicted values and the data any more. This suggests that the very good performance observed on the real data is due to non-trivial signatures in the features of the data. As an additional control, we have performed a decoy classification by permutation of class labels. The AUC also dropped to approximately 0.5 confirming the existence of signatures in the datasets.

### Feature selection revealed important features for each adaptation

In order to assess the importance (predictive power) of each feature for each of the three classification cases, we determined the most discriminative features using the feature selection algorithm of RF. Codes used for feature names are listed in Additional file 7.

Although all features were computed from proteome sequences, the most significant ones tended to be those pertaining to protein sequence composition, providing indirect information about the protein structure.

Initially, the ten most important features were computed and their distributions presented as box-and-whisker plots (Additional file 4). Then, those features were identified that are unique for each classification case. These features are presented below and discussed in together with the remaining features identified and discriminative.

### Domain of life

**Figure 1:**
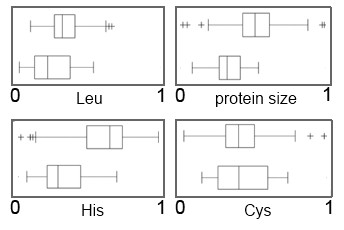
**Four unique features used for classifications regarding domain of life revealed by the feature selection algorithm of RF**. Pairs of box-and-whisker plots are shown for each feature: Leu content, average protein size in a proteome, His content, and 10-Cys content. Box-and-whisker plots represent bacteria and archaea from top to bottom. The feature values are normalized from 0 to 1 from left to right. (+) signs represent outliers.

The frequencies of highly polar and charged amino acids were among the most important features for the classification with regard to the domain of life. Among the most important features unique to this classification problem (Figure 1), bacteria appear to have significantly more His residues than archaea. In addition, the slightly increased Leu content in bacteria has been revealed as a highly discriminative feature, as well as a wider range of possible Cys content in bacteria. Finally, archaeal proteomes are characterized by a decreased protein length. In addition, among features shared between all classification problems (Additional file 4) bacteria appear to have significantly more Gln and a decreased amount of Glu residues than archaea. Although found to have slightly more charged proteomes, bacteria seem to have a decreased content of negative charges.

### Halophilicity

**Figure 2:**
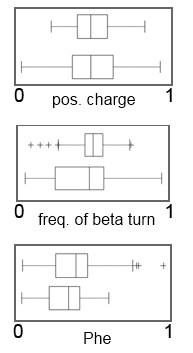
**Three unique features used for classifications regarding halophilicity revealed by the feature selection algorithm of RF**. Pairs of box-and-whisker plots are shown for each feature: positive charge, normalized frequency of beta turn, and Phe content. Box-and-whisker plots represent non-halophiles and halophiles from top to bottom. The feature values are normalized from 0 to 1 from left to right. (+) signs represent outliers.

Among the dominant features that distinguish halophiles from non-halophiles were the frequency of acidic amino acid residues, and the proteome charge. Among the features unique to the classification according to halophilicity (Figure 2) is a decreased content of Phe residues which is a property of halophilic proteomes. Moreover, features such as positive charge and the normalized frequency of beta turn also appeared with high importance with a wider distribution of the feature in halophiles. Among other features that contributes to this classification (Additional file 4), halophiles seem to have almost 2 times more acidic amino acids (especially Glu) than non-halophiles and, as a consequence higher polarity, and higher proteome charge. Furthermore, the Asp composition is increased in halophiles, which is in accord with a general increase in polarity of halophilic proteomes.

### Termophilicity

**Figure 3:**
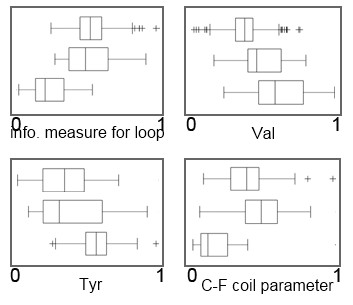
**Four unique features used for classifications regarding thermophilicity revealed by the feature selection algorithm of RF**. Triplets of box-and-whisker plots are shown for each feature: information measure for loop, Val content, Tyr content, and Chou-Fasman parameter of the coil conformation. Box-and-whisker plots represent mesophiles, mesothermophiles and thermophiles from top to bottom. The feature values are normalized from 0 to 1 from left to right. (+) signs represent outliers.

The frequencies of amino acids Val and Tyr are among the unique features to predict thermophilicity (Figure 3). Structural features pertaining to protein secondary structure are also recognized to be important. The information measure for loop is decreased among thermophiles with respect to mesophiles and mesothermophiles. Among the ten most important features (Additional file 4), Gln content is decreasing from mesophilic towards the thermophilic proteomes. On the other hand, Glu and non-polar Val residues content increase from the the mesophilic to thermophilic proteomes. Tyr content is somewhat lower in mesophiles and mesothermophiles in comparison to thermophiles. Asp, on the other hand, displays increased content in mesophiles and mesothermophiles with respect to thermophiles. Generally, negative charge is lower in mesophiles with respect to mesothermophiles and thermophiles. In addition, mesothermophilic and thermophilic proteomes have higher hydrophilicity. Also, the normalized frequency of extended structures is increasing from mesophiles towards thermophiles and the Chou-Fasman parameter of the coil conformation is decreased in thermophiles.

## Discussion

Different environmental conditions impose natural selection and cause adaptive changes among the species. Protein sequence and structure are certainly among the phenotypic properties that can be used by the organism to adapt to the conditions of the environment. It is conceivable that the protein composition and structure are fine-tuned to the physico-chemical conditions of the environment to which they have adapted.

Here, we revealed that the environment in which a species lives can be predicted from the proteome sequence. We have studied correlations between the environmental niche of a species and 42 physico-chemical properties derived from the amino acid composition of the proteomes. The supervised classification algorithms (RF and SVM) could very accurately distinguish bacteria from archaea, halophiles from non-halophiles, as well as thermophiles and mesothermophiles from mesophiles. They also allowed identifying the ten most important physico-chemical proteome features for each environmental adaptation, leading to mechanistic and functional insight.

Note that distributions of individual features overlap between the different environments, even for the most relevant features (Figures 1, 2 and 3), meaning that no single feature may be sufficient to accurately discriminate between the classes. However, with a capable computational apparatus and a sufficient sample size, it becomes feasible to discover also the less obvious connections between multiple proteome physico-chemical characteristics and the environment. In other words, each of the features influences the probability of a proteome belonging to a certain environment. The individual features are an important part of the 'signature' of environmental adaptation and can be interpreted as such - but a larger number of them is necessary to achieve an accurate classification (high AUC scores from Table 1).

We used two fundamentally and conceptually different classification algorithms (RF and SVM). RF is basically a collection of decision tree classifiers [13], which try to describe the relationship of the features and the class in terms of sets of nested rules (trees), such as e.g. "if value of feature f is below/above threshold value t, the proteome belongs to a thermophile/halophile/mesophile etc." On the other hand, SVMs are algorithms based on statistical learning theory, which find the hyperplane that separates the data points (here: proteomes) of different classes so that the width of the margin between the points of different classes is maximized [15]. Both of these approaches may generate non-linear models, which has the following implications to the issue at hand: (a) relationships between a proteome feature and a class variable will be captured even if they are non-monotonic; a hypothetical example illustrating this would be e.g. that thermophiles might have either very high, or very low values of a certain feature, while mesophiles span the range in between; and (b) if two or more proteomic features do not correlate to the class by themselves, but become informative when combine, such relationships will be captured and used to deduce the class; here, a hypothetical example might involve thermophiles which have both low values of feature f and high values of a feature g at the same time, but not vice versa. The results were strikingly similar between the two different classification methods (RF and SVM). This strongly suggests that the identified proteome signatures are biologically relevant and not mere artifacts of over-fitting or the algorithms used. To our knowledge, this is the first report of successful classification of three environment-related problems based on the same set of features.

### Domain of life

The variation of proteomic properties between domains of life has previously been addressed. Kaoru et al. [16] successfully constructed a tree of life based on protein domain organization. Furthermore, Pe'er et al. [17] detected correlations between the domain of life and oligopeptide compositions, while Tekaia et al. [18] used correspondence analysis and amino acid composition to obtain high classification performance when distinguishing eukaryotes from prokaryotes. Both in the study by Tekaia and in the present work, the His content and generally the content of polar and charged amino acids, was an important feature distinguishing between different domains of life. Moreover, we found that archaea seem to have proteomes enriched in negatively charged amino-acids. This adaptation is probably crucial at the protein surface where dipole-dipole interactions are replaced by stronger electrostatic ones in order to stabilize the protein surface.

We also showed significant differences in other features between bacteria and archaea. Normalized frequency of extended structure [19] is lower in bacteria than archaea. Together with the finding that archaea have shorter polypeptide chains, this may indicate that atchaeal proteins have a tendency to be more compactly packed. While it is challenging to give a reason for this, the peculiarity of archaeal niches and lifestyles could contribute to the general difference in protein size [20,21].

It is extremely hard to distinguish adaptation signatures from phyla signatures when classifying archaea from bacteria. Archaea with fully sequenced genomes thrive in a wide range of extreme environmental conditions. Therefore, we cannot ignore that the discrimination of bacteria from archaea, and the corresponding feature selection, may reflect partially an adaptation to different environments. However, the availability of a large number of bacterial proteomes used in this analysis, especially from mesophilic conditions, allows to reduce this problem and to perform a more precise classification and feature selection.

### Halophilicity

Halophiles are organisms that thrive in highly salty habitats, such as salt lakes or salterns. The concentration of salt in their cytosol can reach as high as 4 M, which is challenging for macromolecules from both a structural and functional point of view. We have revealed protein features that these organisms have evolved in order to maximize protein stability in saline conditions.

We found that halophilic proteomes are generally characterized by a decreased charge, a higher proportion of acidic residues, and higher hydrophilicity with respect to non-halophilic proteomes. In addition, higher glutamate and aspartate content and somewhat lower glutamine content are among the specificities of halophilic proteomes. Generally, Glu and Asp residues contribute to the solubility of proteins and could therefore be favored in proteins from halophilic environments [20]. Based on our results it would be possible to speculate that Glu could be more import - ant than Asp in order to achieve the acidity of the proteome. This may be due to the fact that Glu has the highest capacity to bind water molecules, a property highly important in the state of osmotic shock [22].

Furthermore, the role of structure-related parameters that were shown to be important for the adaptation to high salt concentrations was considered. The normalized frequency of beta turns was shown to be important descriptors of halophilicity. Halophiles have a wider distribution of possible contents of amino acids with a high propensity to form beta-turns. This might suggest that beta turns are unfavorable structures in halophiles, possibly due to their increased flexibility that may reduce protein stability under the denaturing conditions of high salinity.

A bias in amino acid composition has previously been detected in halophiles. An increased amount of acidic residues has been described, including an increased ratio of acidic (Glu and Asp) to basic amino acids, resulting in a lower isoelectric point [23-28]. Also, a drastic drop in lysine content has been pointed out as a property of halophilic adaptation [29]. This can result in increased polarity and charge at the surface of a halophilic proteins [30]. While the cores of halophilic proteins have been shown to not significantly differ from mesophilic ones, surface properties repeatedly appeared as contributing to protein stability under high salt concentrations. Consistently with our results, aspartic acid, lysine, asparagine, alanine, and threonine have previously been identified as the residues that account for the most important differences between halophiles and mesophiles. While our study could reproduce these previous results, it also detected new important features that may play a role in adaptation to high salt concentrations.

### Thermophilicity

Thermophiles are commonly defined as organisms with an optimal growth temperature above 55°C, with facultative thermophiles being able to survive both below and above 55°C [31]. Rather than just looking at the optimal growth temperature, we propose to use the temperature *range *in which a species can survive as a more accurate measure for thermophilicity. Thus, in addition mesophiles and thermophiles, having their entire optimal temperature ranges in mesophilic and thermophilic ranges, respectively, we have defined a class of mesothermophiles whose range begins in the mesophilic temperature range and extends to the thermophilic one.

Numerous studies performed on thermophilic proteins have shown that there is no single mechanism of adaptation to high temperatures. Proteins of thermophilic organisms are generally considered highly stable. We have found that thermophilic proteins are rich in Val and Tyr residues that may be able to promote tight packing of the hydrophobic core and hence increase the overall stability. The increased polarity of mesothermophilic and thermophilic proteomes relative to mesophilic ones contributes to the increased stability of the protein surfaces by increasing the number of polar contacts. Furthermore, flexible structures, such as loops, seem to be unfavorable as the amino acid residues that promoted their formation are not abundant. On the other hand, extended structures, such as beta sheets are favorable among thermophiles.

It has previously been found that the residues forming thermophilic protein cores are mostly conserved, indicating their primary role in protein stabilization. Stabilizing interactions, however, are often also found in the less conserved parts of thermophilic proteins. This includes an increased number of ion pairs (Arg, Lys, Glu, Asp) at the surface and a decreased number of exposed hydrophobic surfaces [32]. More specifically, an increase in charged residues, at the expense of polar uncharged ones, has been found [33]. Ratios of these amino acids have previously been shown to be important for protein flexibility [34].

Tekaia et al. [8] have performed correspondence analysis on 56 prokaryotic and eukaryotic proteomes in order to extract relevant characteristics of the lifestyle and evolutionary trends of these species. The amino-acid composition of the 56 proteomes was considered a property that may enable discrimination between species. Indeed, they were able to distinguish between mesophiles, thermophiles, and hyperthermophiles, irrespective of the domain of life they belong to. The authors have further found an increasingly high GC content with increasing optimal growth temperature.

Additionally, Zeldovich et al. [35] have examined whether selection on amino acid usage shapes the characteristics of genomic DNA sequences in thermophiles. They found the amino acids IVYWREL as those whose total frequency in a proteome most strongly correlates with the optimal growth temperature of the organism. Their method is in essence a special case of multiple linear regression (MLR) on amino acid frequencies, where the coefficients are constrained to either 1 (amino acid correlates) or 0 (does not correlate). Our approach contrasts Zeldovich et al. in two points: First, the RF and SVM classifiers are well-suited for situations where the optimal growth temperature is non-linearly correlated with the features, or where the features become informative only in certain (non-linear) combinations. The second point concerns the features used to describe proteome composition. We opted for summary statistics of commonly used physico-chemical properties of amino acids. This provides a more complete (and possibly more informative) description of the proteomes than just considering amino acid frequencies alone. In addition, our description allows a more direct interpretation of the biophysical adaptations that a proteome undergoes as it adapts to high temperatures.

It must be noted that our current analysis works with average values of proteomes' physico-chemical features, while it does not explicitly account for the shape of a feature's distribution among proteins within a proteome, such as e.g. a feature's distribution tail length, or presence of outlier proteins, or similar. A deeper insight into what differentiates the proteomes of a certain environment might be gained by using a richer description of these within-proteome distributions instead on analyzing only the distributions' central tendencies. After having briefly explored the distributions' shapes within a few representative proteomes (provided in Additional file 5), it would seem this is indeed a desirable venue for future investigations.

## Conclusions

We applied two fundamentally different machine learning methods, support vector machines (SVM) and random forests (RF) to successfully address three different classification cases: to distinguish bacteria from archaea, halophiles from non-halophiles, as well as mesophiles from thermophiles and mesothermophiles, always by using a single set of 42 features. Feature selection has revealed most important features that reflect best each adaptation: proteome charge and average protein length for bacteria vs. archaea; beta-turn content and positive charge for halophiles vs. non-halophiles; protein compactness and content of disordered structures for thermophiles vs. mesothermophiles vs. mesophiles. So far, this is the first study pointing out that prokaryotic proteomes carry signatures of their environmental niches and offers a possibility of environmental niche prediction from the protein sequence, all based on the same set of features.

## Methods

### Data collection

We have collected 1107 (1025 bacteria and 82 archaea) prokaryotic proteomes in order to study the adaptation of proteomes regarding the domain of life. To construct a dataset to pursue the study of adaptations to high temperature and high salinity, we have selected a total of 192 prokaryotic (153 bacterial and 39 archaeal) proteomes based on the availability of environmental niche descriptors at the time of data collection. The collected proteomes were freely available from the High-quality Automated and Manual Annotation of Microbial Proteomes (HAMAP) database or from the National Center for Biotechnology Information (NCBI) [36]. Moreover, we harvested various databases and literature sources to collect information about the environment where each organism lives. This included the growth temperature range and the NaCl concentration range. Our dataset consists of 103 mesophilic species and 89 extremophilic species (thermophiles and halophiles). We have given three different class labels to each instance (species) in our dataset, encoding the domain of life they belong to (archaea or bacteria), the temperature range they tolerate (mesophile, mesophiles/thermophile, or thermophile), and the NaCl concentration they live in (non-halophile or halophile). All classifications were done using the same set of features.

### Selection and computation of proteome features

In addition to the amino acid composition of each proteome, we have selected 48 biologically interpretable features out of the 54 features described in Atchley et al [37]. In addition to this set of features, we also included the isoelectric point [38] and the protein length. Furthermore, we have defined 8 features that represent ratios of frequencies (f) of different amino acids types: f(charged)/f(non-charged), f(charged)/f(all), f(polar)/f(non-polar), f(polar)/f(all), f(disorder-promoting)/f(order-promoting), f(disorder-promoting)/f(all), f(negatively charged)/f(positively charged), and f(negatively charged)/f(all).

We have shortened this list of proteome features so as to reduce redundancy between the remaining features. First, we have computed the rank correlation coefficients for all pairs of features, within all 192 proteomes in the dataset. Then, we have performed hierarchical clustering of features based on the absolute value of the rank correlation coefficient (as described in Additional file 6); the agglomeration method used in the clustering was 'unweighted pair-group average' and the correlation threshold was set to 0.9. Finally, we have selected one representative feature per cluster, the one closest to the center of the cluster. The features were computed for each individual protein within a proteome. The values for the entire proteomes were obtained by averaging over all proteins.

### Classification of species

We have used two fundamentally different algorithms for the three classification cases: Random Forests (RF) and Support Vector Machines (SVM) [12,13]. Classification accuracies are presented as ROC curves plotted in Matlab using the votes from the RF and the probability outputs from the SVM, respectively. The list of codes used for feature names is given in the Additional file 7.

### Support Vector Machines

SVMs are a class of algorithms based on statistical learning theory, which find the hyperplane that separates the data points (here: proteomes) of different classes so that the width of the margin between the points of different classes is maximized; wider margins imply lower generalization error. Additionally, application of the so-called 'kernel trick' - use of a specialized non-linear function (commonly a Gaussian function) to map the data into a very high-dimensional space, allows the SVM to find separating hyperplanes of an arbitrary degree of curvature. In practice, SVMs have been shown to have high classification accuracy in a variety of scenarios, see eg. [39] for a review of SVM usage in computational biology, and applications in chemoinformatics have been reviewed in [40].

We performed SVM classification using the LibSVM software [41]. The original data set was divided into three training and three testing sets by random stratified selection without replacement. The training sets consisted of two thirds of the total number of instances and the test sets comprised the remaining third. All feature values were normalized to the interval 0[1] so that the minimal value of the feature was zero and the maximum was 1 for each class, while the values in between were scaled accordingly. We used ten-fold cross-validation to measure classification accuracy and prevent overfitting. The *C *and *gamma *parameters of the SVM were tuned using the grid search tool of LibSVM and we used radial basis function kernels. We defined three temperature classes: mesophiles (the entire range is in the mesophilic region), meso-thermophiles (the range begins in the mesophilic and ends in the thermophilic region), and thermophiles (the entire range is in the thermophilic region). The resulting three-class problem was reduced to a two-class problem by *one-against-one *and *one-against-all *pairwise classification. The results were better than those obtained using true multiclass classification using Crammer and Singer's formulation as implemented in the BSVM library [42] (data not shown).

As a control for each classification case the feature values were randomly permuted and classification repeated. In addition, we have performed decoy classification by the permutation of class tags.

### Random Forests

The RF algorithm [13] produces an ensemble of decision tree classifiers, where each decision tree is constructed by recursively partitioning the data by feature value tests (forming 'nodes') so as to reduce the entropy of the class label in the resulting partitions ('branches'). The individual trees are trained on bootstrap samples of the dataset, while the final predictions of a RF model are obtained by averaging over all the trees ('voting') to guard against overfitting the data. Additionally, the choice of features at each node is artificially restricted to a subset of the available features to de-correlate the individual trees, which has been shown to benefit the accuracy of RF models [13]. After a RF model has been trained, feature importance can be estimated by permuting the values of a single feature and measuring the prediction error of the RF model before and after the permutation; if the feature was relevant for the discrimination of the classes, the prediction error will rise after permuting the feature's values. A more detailed description of this method can be found in e.g. [43] Note that this approach captures features which correlate to the class non-monotonically, and also the features that are correlated to the class only in combination with other features.

After ten most important features were identified, those features were excluded that show up in all three classification cases. Features unique to each classification case are presented separately and all ten most important features together are in Additional file 4.

Classification of species using RF was performed using the PARF implementation [44] with a forest size of 1000 trees and all other parameters left at default values. Additionally, we extracted the list of most significant features using the PARF's feature selection function. Training, control, and validation were done in the same way as for the SVM (described above).

## Authors' contributions

All authors have read and approved the final manuscript.

ZS: has made substantial contributions to acquisition of data, analysis and interpretation of data, as well as writing of the manuscript

NN: has made substantial contributions to acquisition and interpretation of data

FS: has made substantial contributions to analysis of data and interpretation of data, and writing of the manuscript

TS: has made substantial contributions to analysis of data

IFS: has made substantial contributions to conception, design and interpretation of data

AK: has made substantial contributions to conception, design, acquisition and interpretation of data, as well as writing of the manuscript

## Supplementary Material

Additional file 1 **List of 1107 species used in this study, with values of each feature**. Codes used for feature names are listed in Additional file 7. Click here for file

Additional file 2 **Classification results shown as receiver operating characteristic (ROC) graphs with associated area under the curve (AUC) values, by using SVM**: (A) domain of life classification, (B) halophilicity classification; by using RF: (C) domain of life classification, (D) halophili - city classification. Click here for file

Additional file 3 **Classification results shown as receiver operating characteristic (ROC) graphs with associated area under the curve (AUC) values for temperature adaptation, by using SVM**: (A), mesophiles vs. others (B), mesothermophiles vs. others, (C) thermophiles vs. others, (D) mesophiles vs. mesothermophiles, (E) mesophiles vs. thermophiles, and (F) mesothermophiles vs. thermophiles; by using RF: (G) mesophiles vs. others, (H) mesothermophiles vs. others, (I) thermophiles vs. others, (J) mesophiles vs. mesothermophiles, (K) mesophiles vs. thermophiles, and (L) mesothermophiles vs. thermophiles. Click here for file

Additional file 4 **Summary of feature selection results**. (A) Ten most important features for classifications regarding domain of life revealed by the feature selection algorithm of RF. Pairs of box-and-whisker plots are shown for each feature labeled with a number: 1-Gln content, 2-Leu content, 3-normalized frequency of extended structure, 4-negative charge, 5-average protein size in a proteome, 6-Glu content, 7-charge, 8-His content, 9-ratio of charged and non-charged amino acids, 10-Cys content. Box-and-whisker plots represent bacteria and archaea from top to bottom. (B) Ten most important features for classifications regarding halophilicity revealed by the feature selection algorithm of RF. Pairs of box-and-whisker plots are shown for each feature labeled with a number: 1-negative charge, 2-charge, 3-hydrophilicity value, 4-positive charge, 5-Gln content, 6-Glu content, 7-ratio of charged and non-charged amino acids, 8-normalized frequency of beta turn, 9-Asp content, 10-Phe content. Box-and-whisker plots represent non-halophiles and halophiles from top to bottom. (C) Ten most important features for classifications regarding thermophilicity revealed by the feature selection algorithm of RF. Triplets of box-and-whisker plots are shown for each feature labeled with a number: 1-Gln content, 2-information measure for loop, 3-Glu content, 4-Val content, 5-normalized frequency of extended structure, 6-hydrophilicity value, 7-Tyr content, 8-Asp content, 9-negative charge, 10-Chou-Fasman parameter of the coil conformation. Box-and-whisker plots represent mesophiles, mesothermophiles and thermophiles from top to bottom. In all plots feature values are normalized from 0 to 1 from left to right. (+) signs represent outliers. Click here for file

Additional file 5 **Histograms showing shapes of distributions of features' values within six representative proteomes: a mesophilic, thermophilic and halophilic bacterium, and a mesophilic, thermophilic and halophilic Archaeon**. Click here for file

Additional file 6 **Pairwise correlation coefficients within the original set of 79 proteome features, and a visualization of the hierarchical clustering of these features**. Applying a threshold (rank correlation < 0.9) to the clustering yielded 42 feature clusters whose representatives were chosen as the final, reduced-redundancy 42 feature set. Click here for file

Additional file 7 **Final list of 42 used features in the study, together with their codes**. Click here for file
