# Supplementary figures and images for: Proteome sequence features carry signatures of the environmental niche of prokaryotes
Source: BMC Evol Biol. 2011 Jan 26;11:26. doi: 10.1186/1471-2148-11-26 (PMC3045906; doi:10.1186/1471-2148-11-26)

A

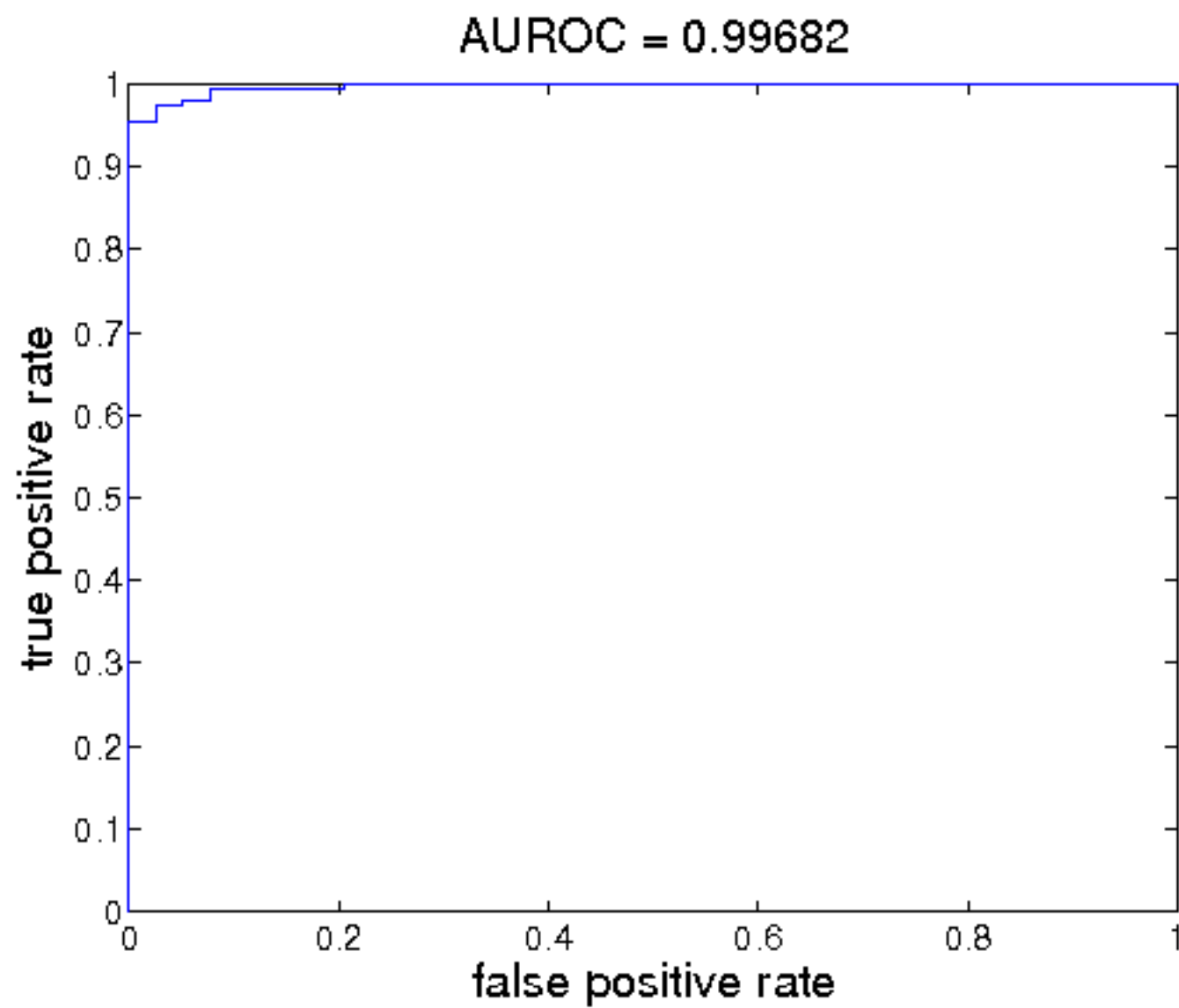

B

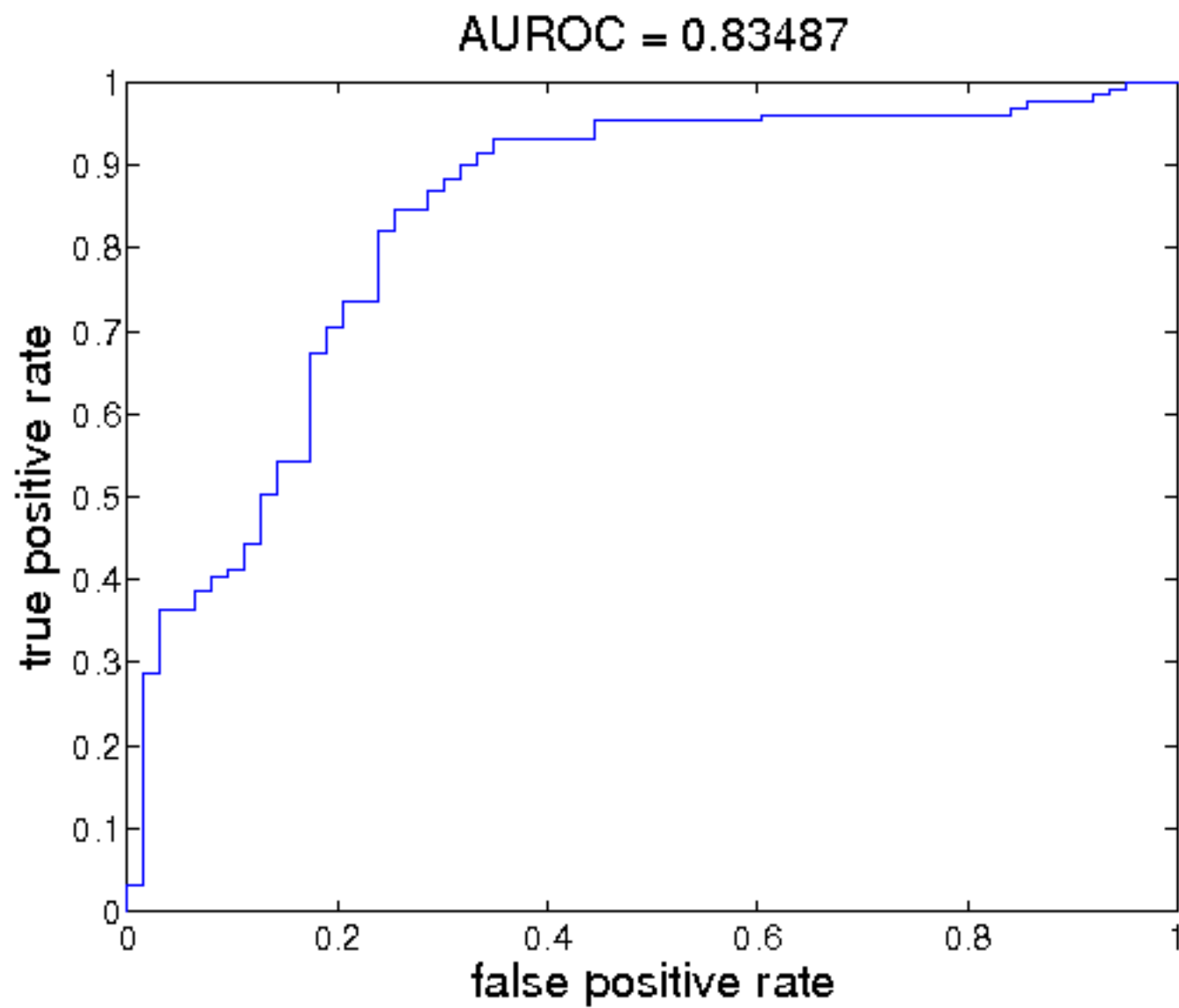

C

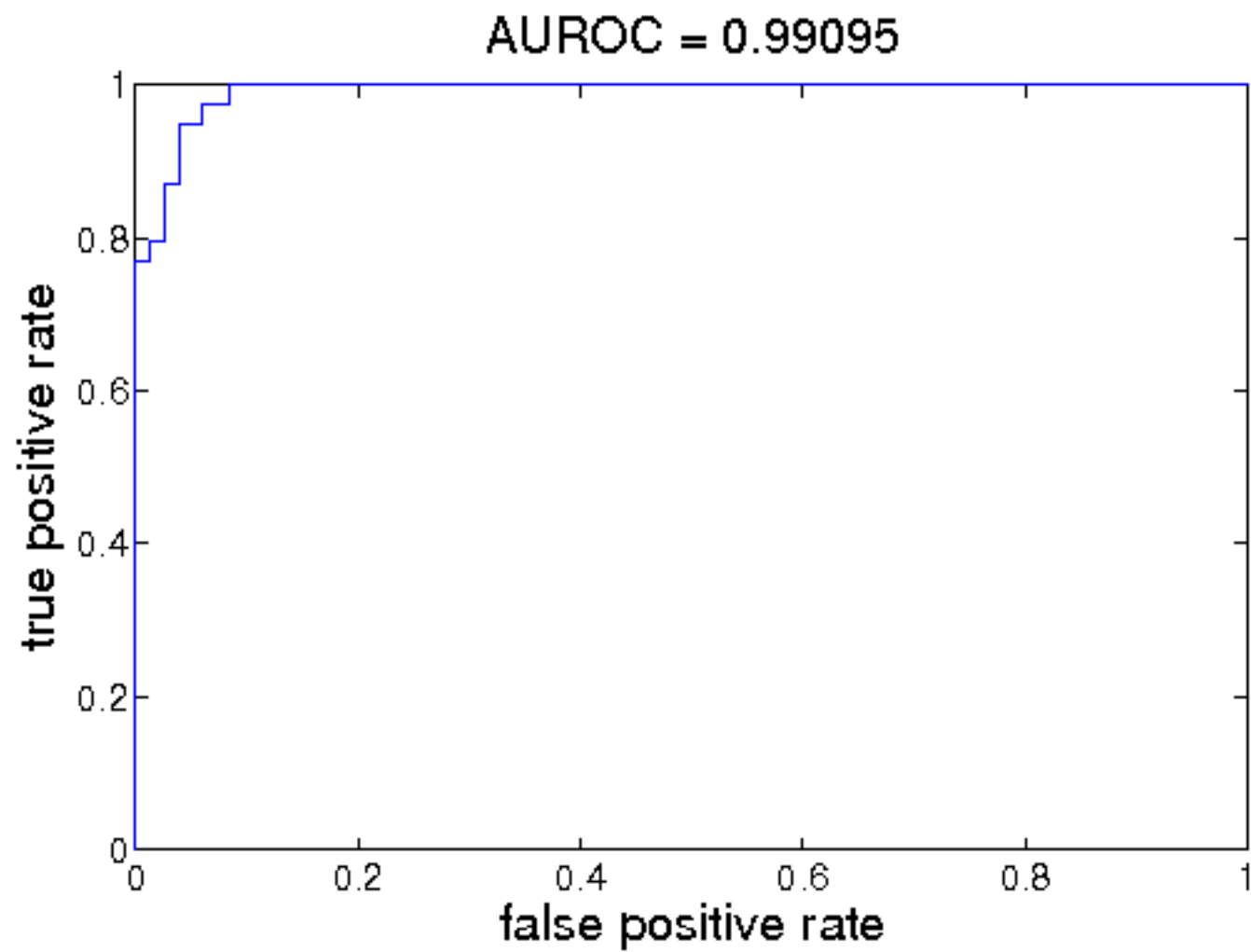

D

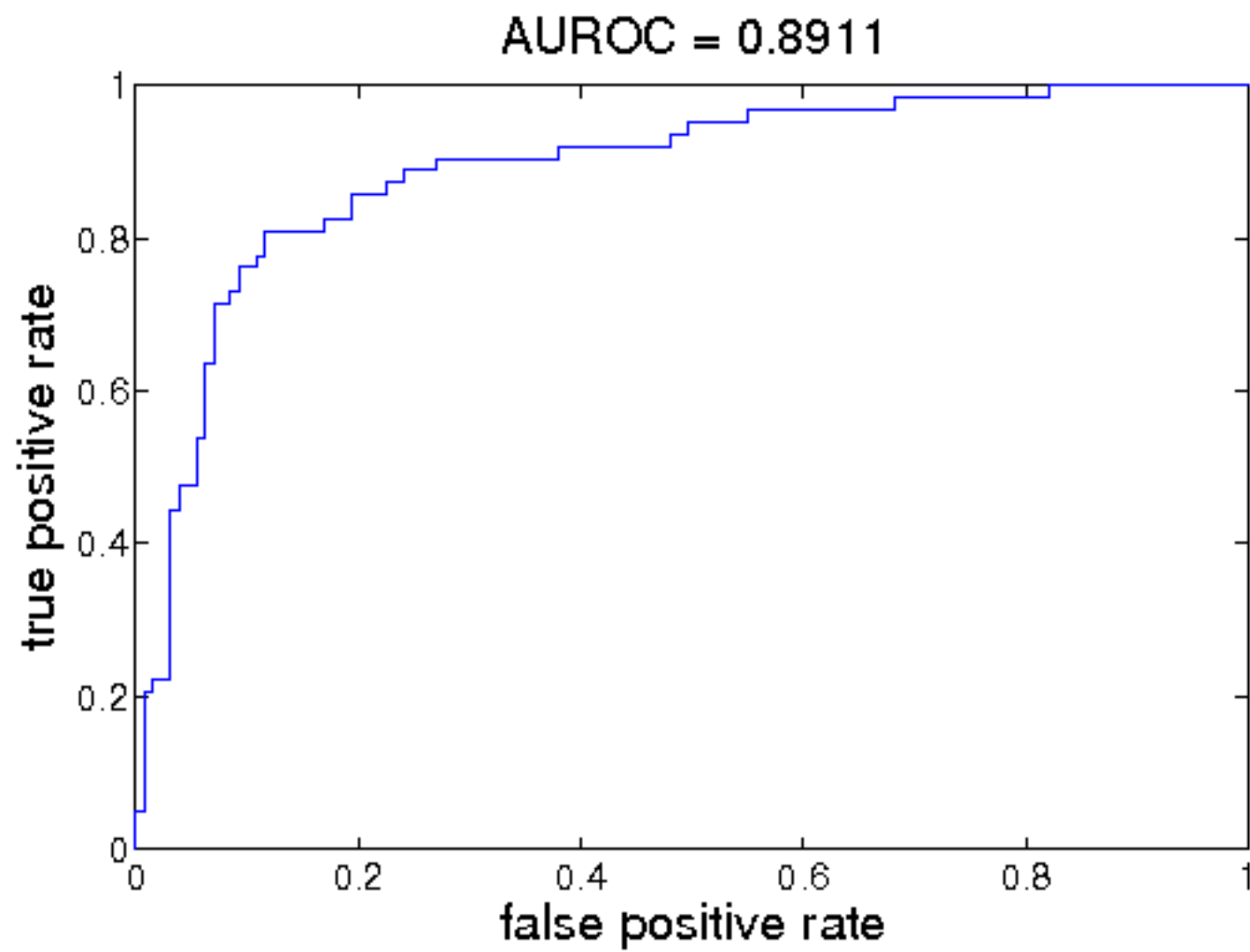

Supplement: Additional file 2 — Classification results shown as receiver operating characteristic (ROC) graphs with associated area under the curve (AUC) values, by using SVM: (A) domain of life classification, (B) halophilicity classification; by using RF: (C) domain of life classification, (D) halophili - city classification. [file 1471-2148-11-26-S2.PDF]

A

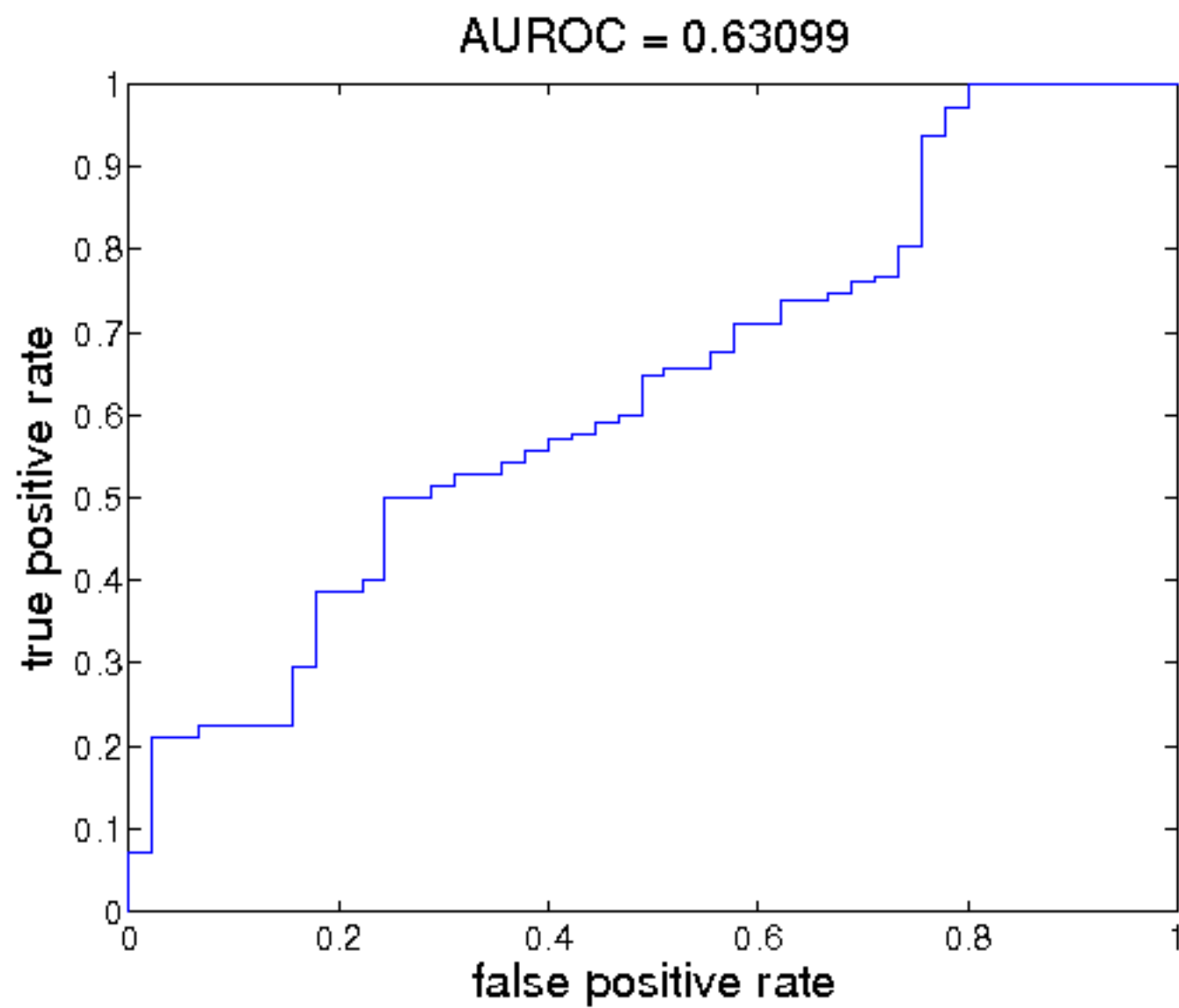

B

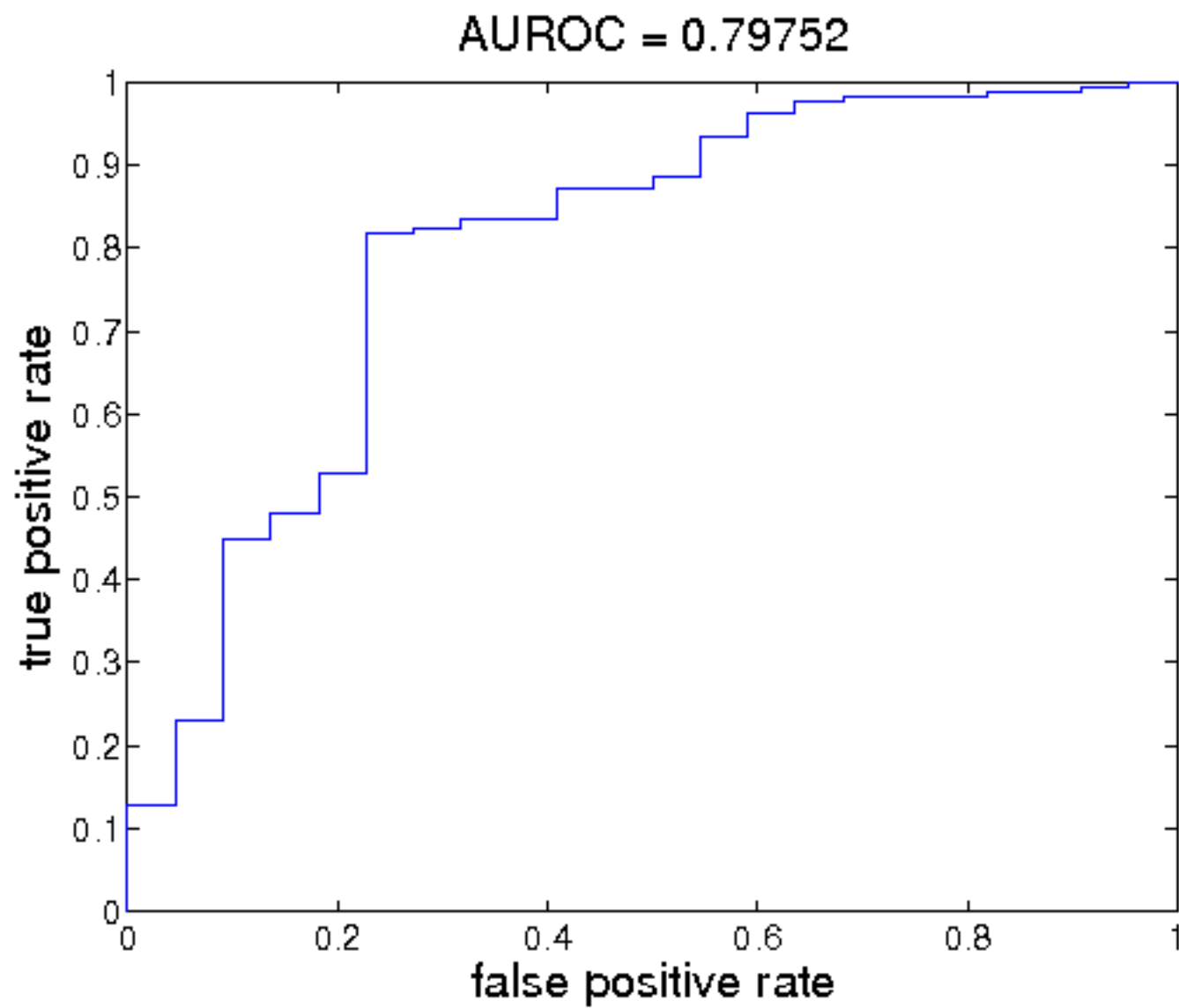

C

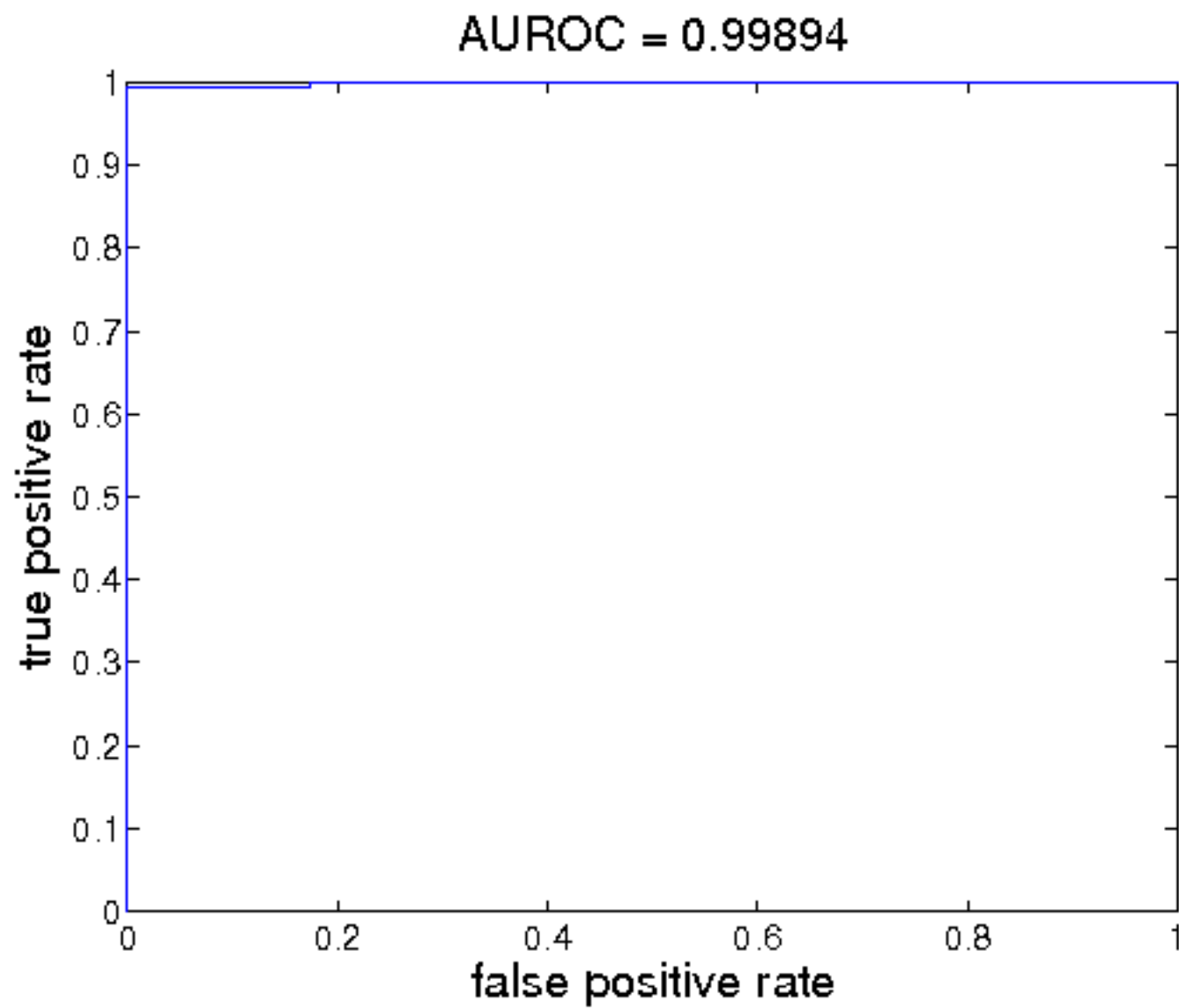

D

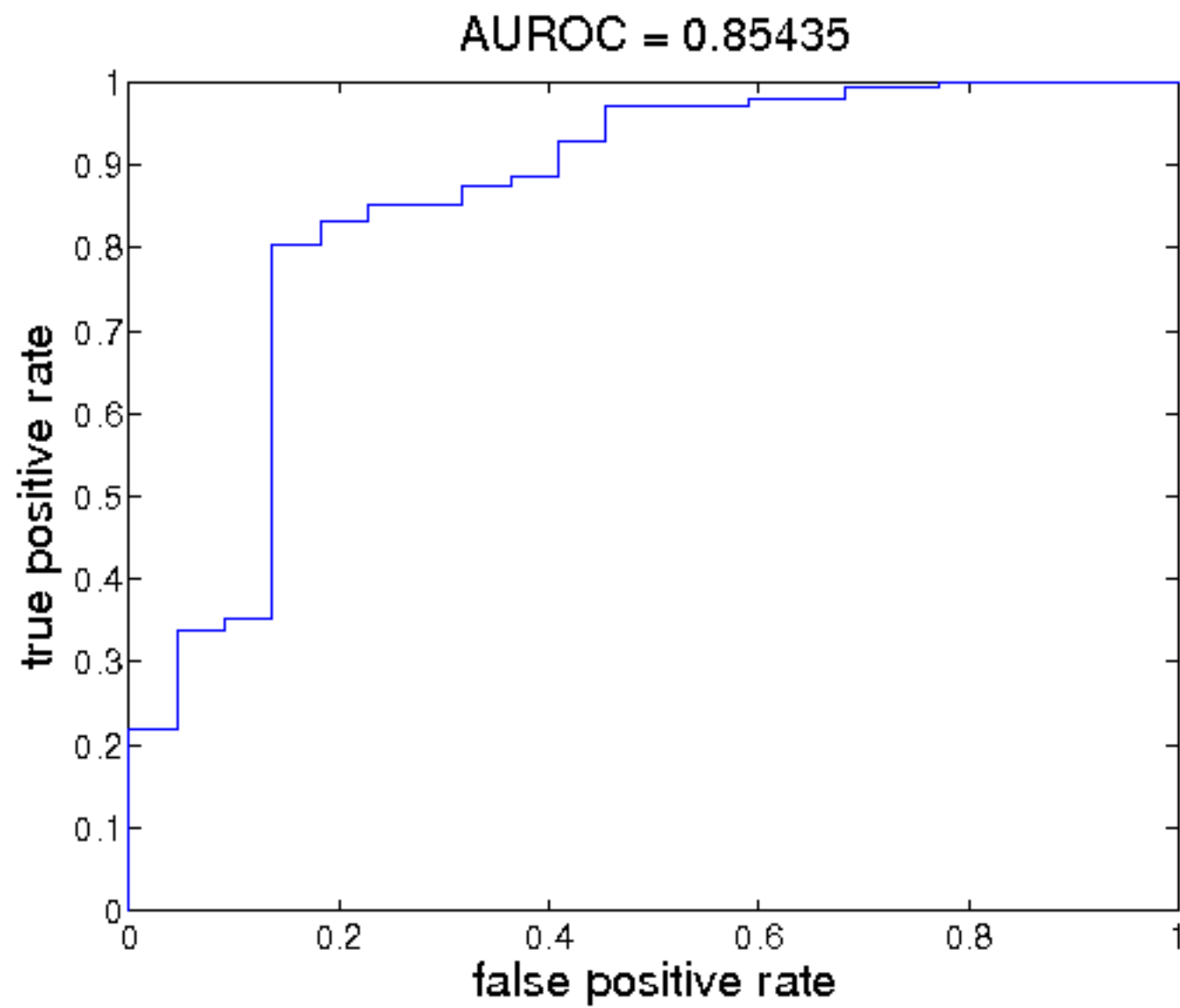

E

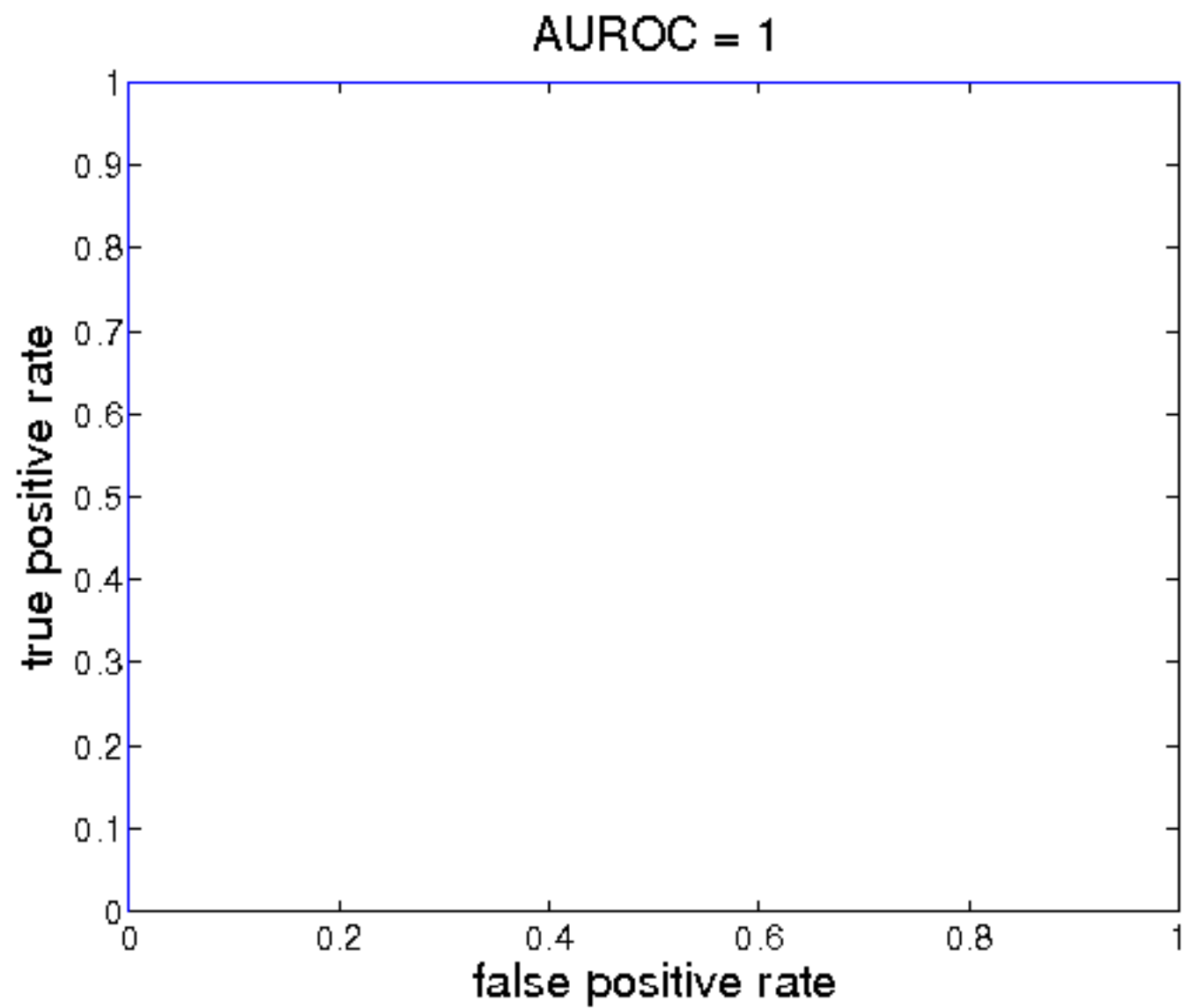

F

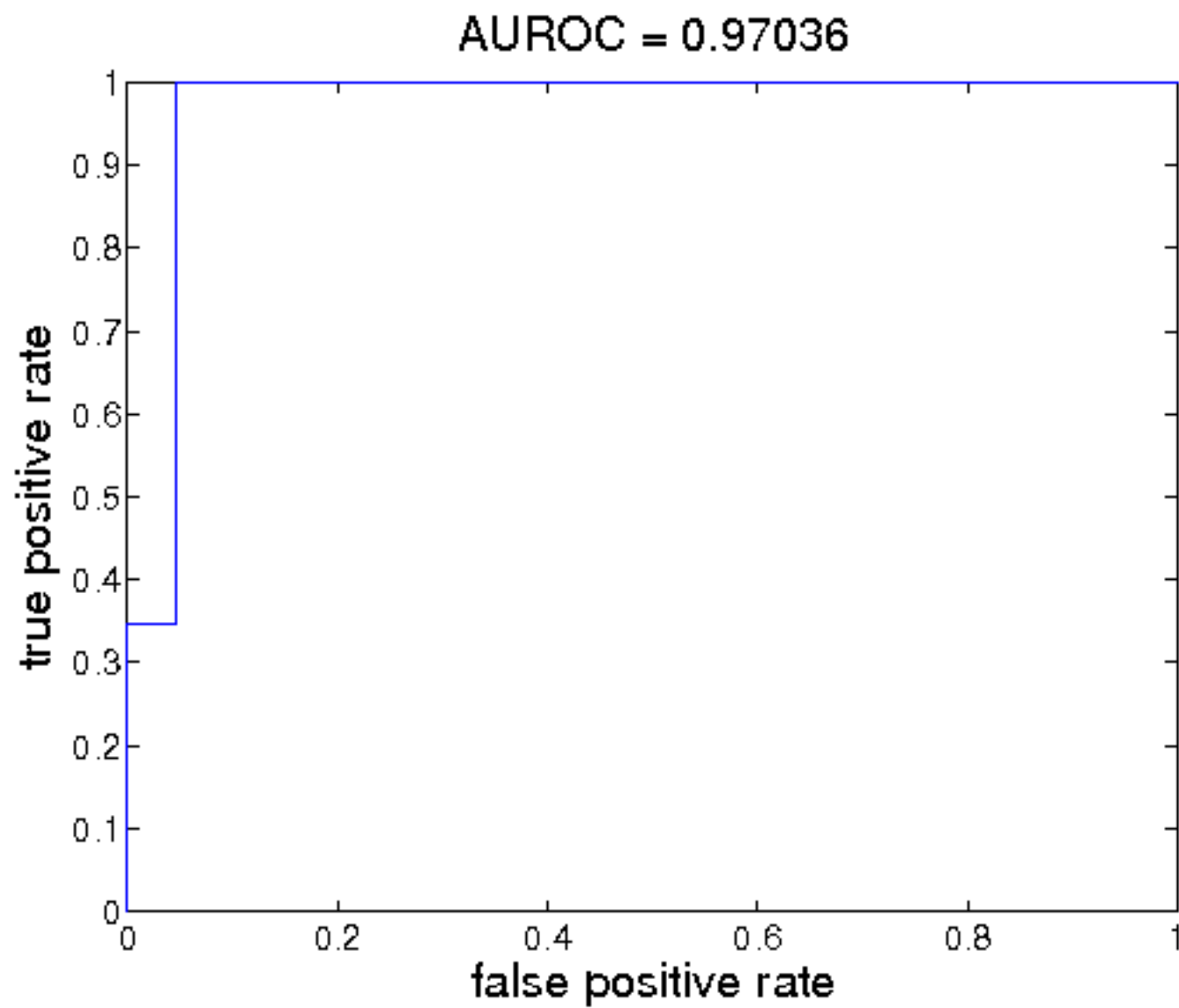

G

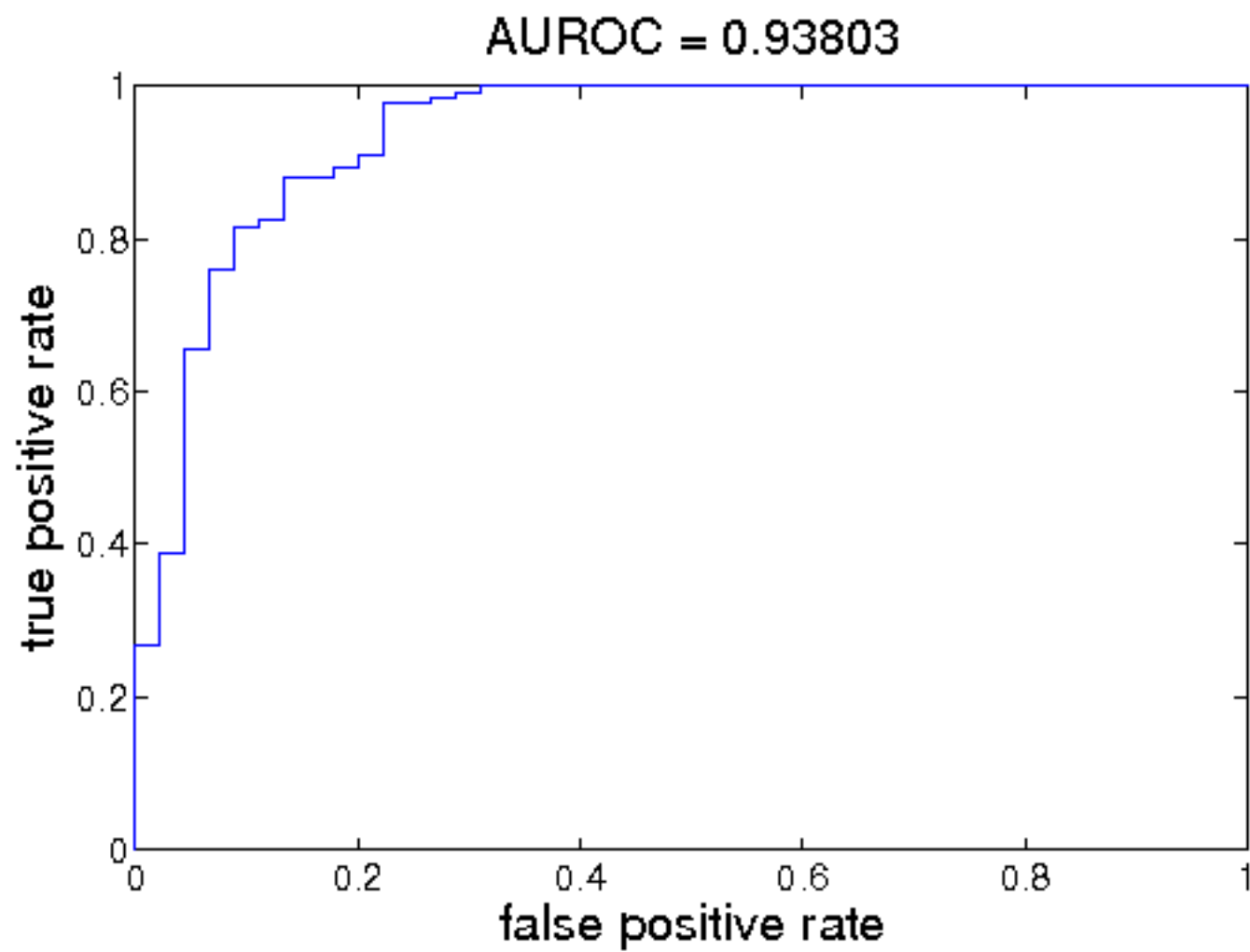

H

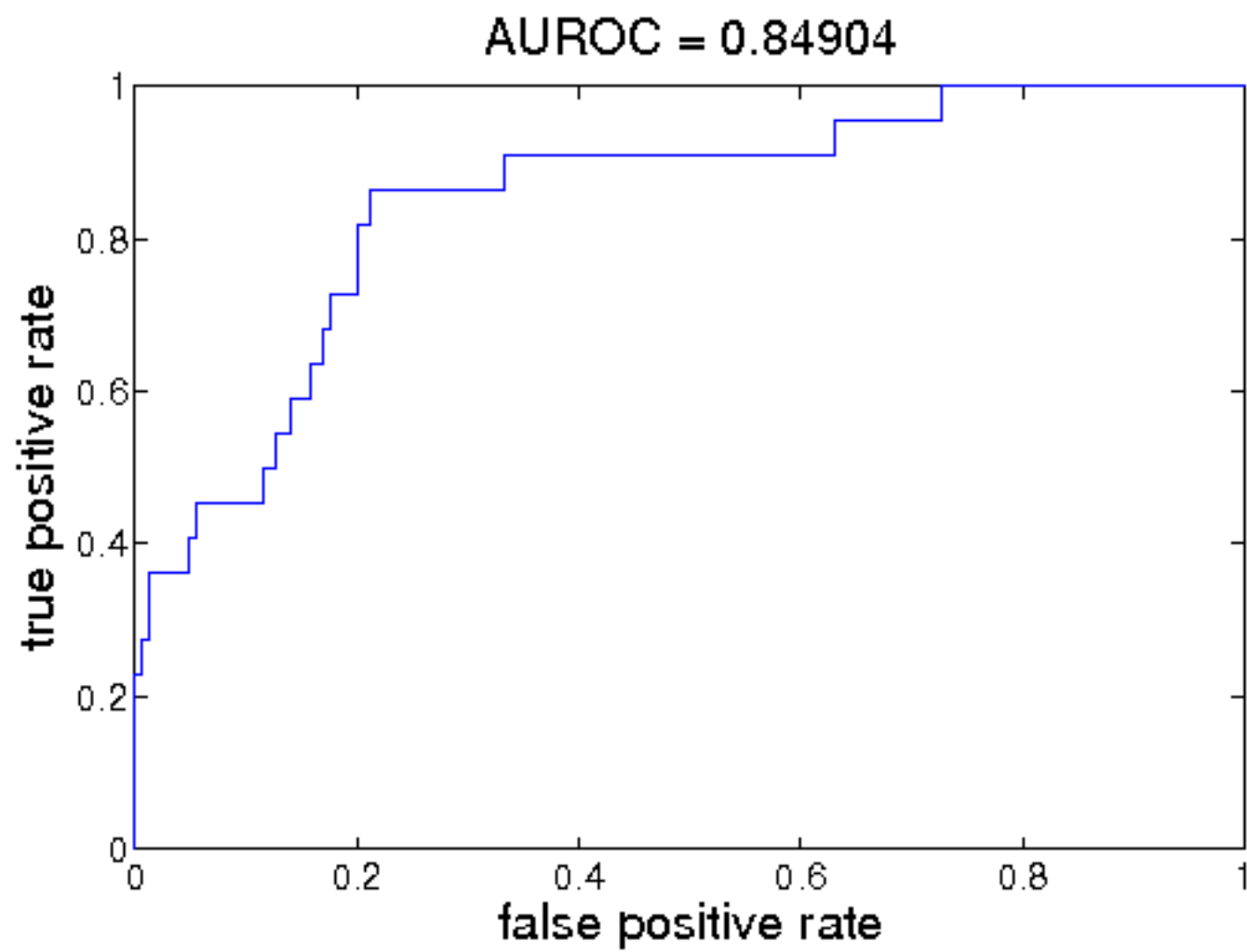

1

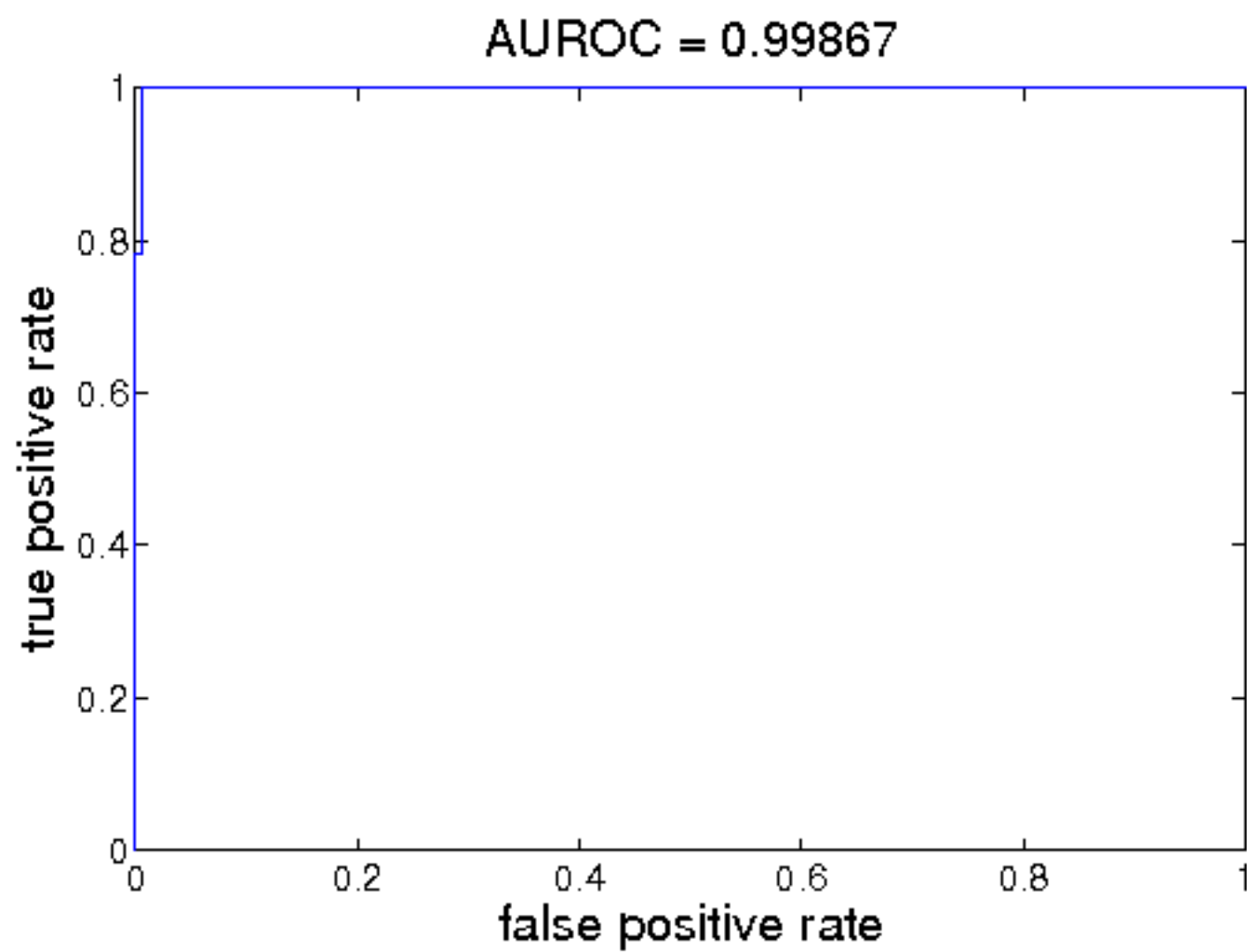

J

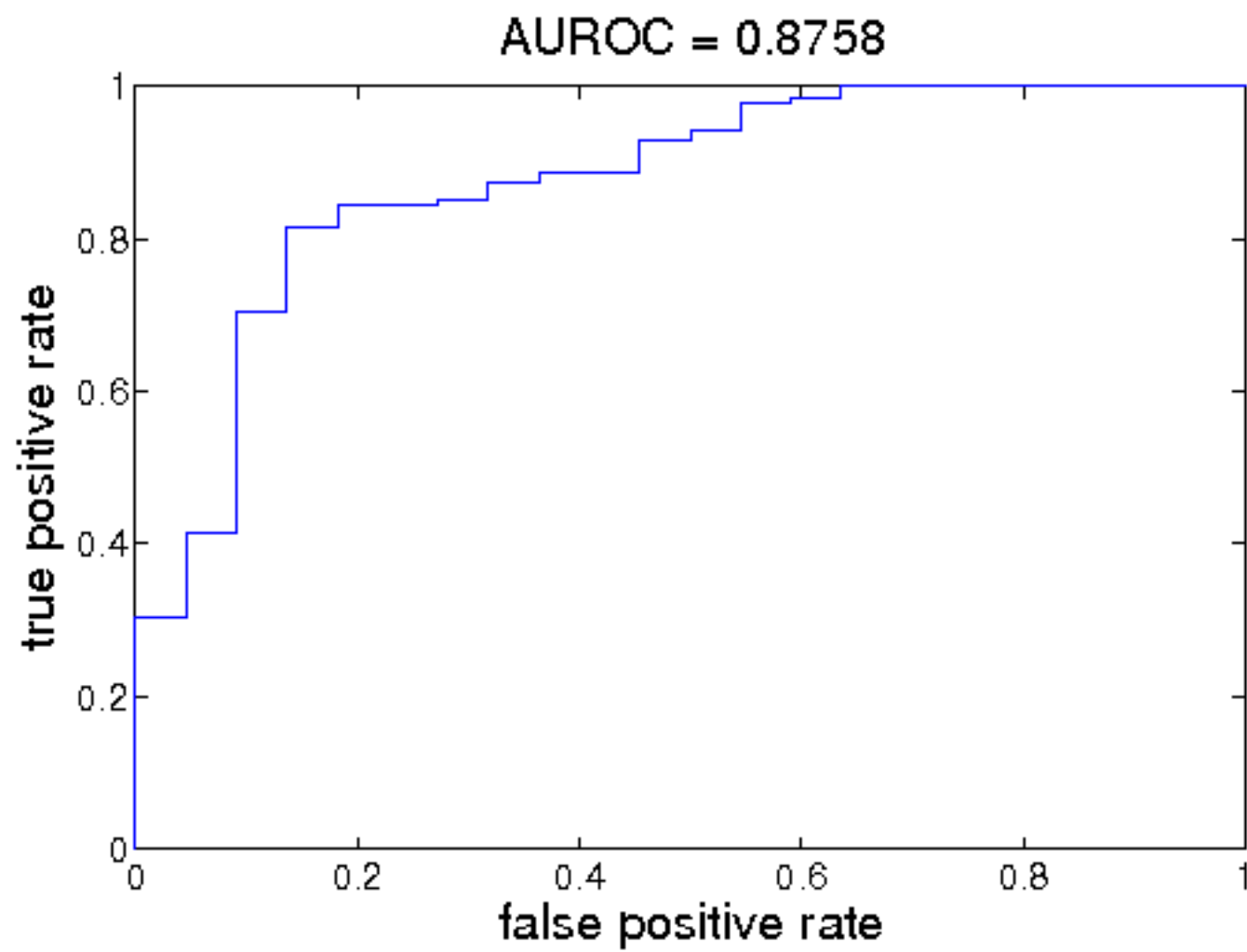

K

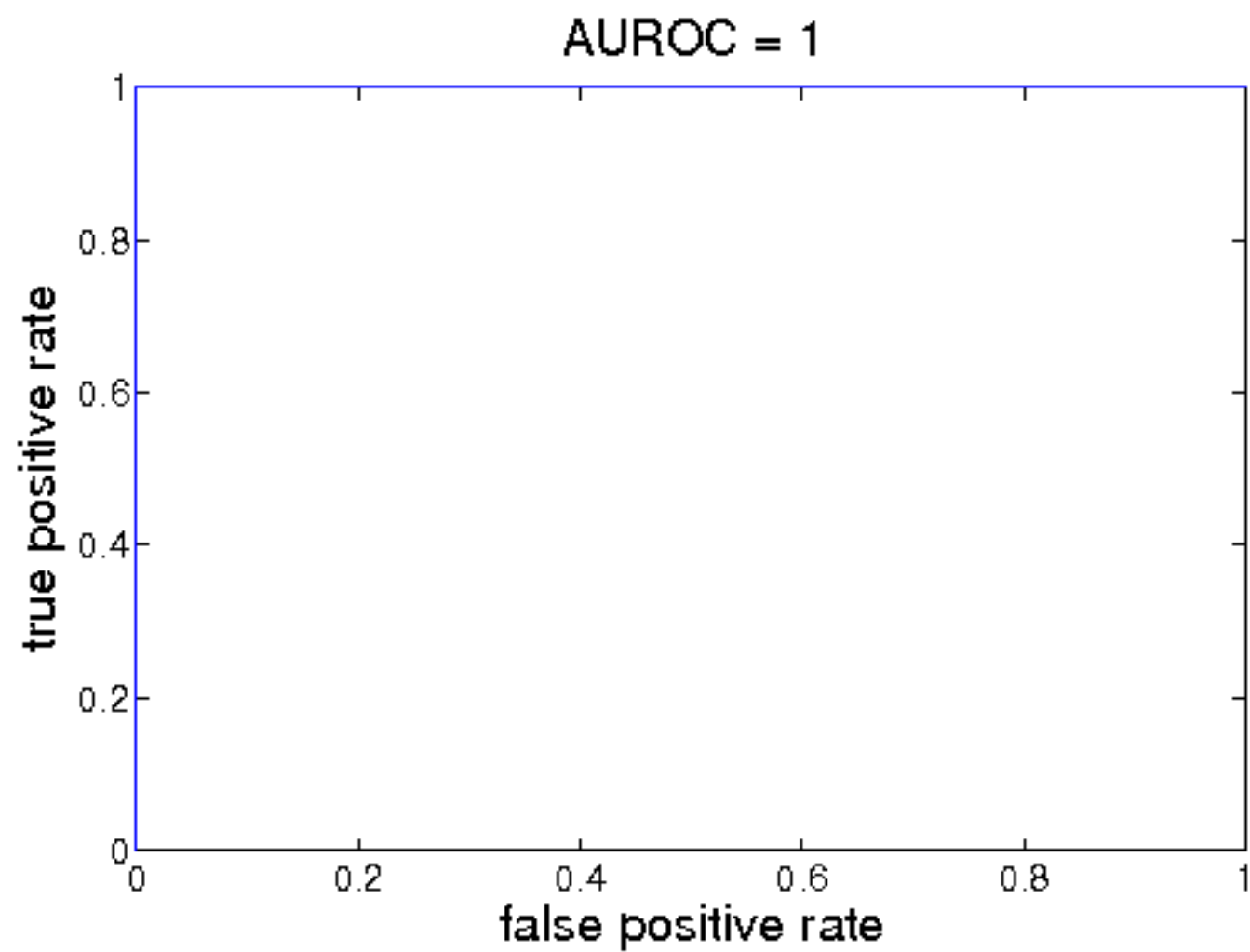

L

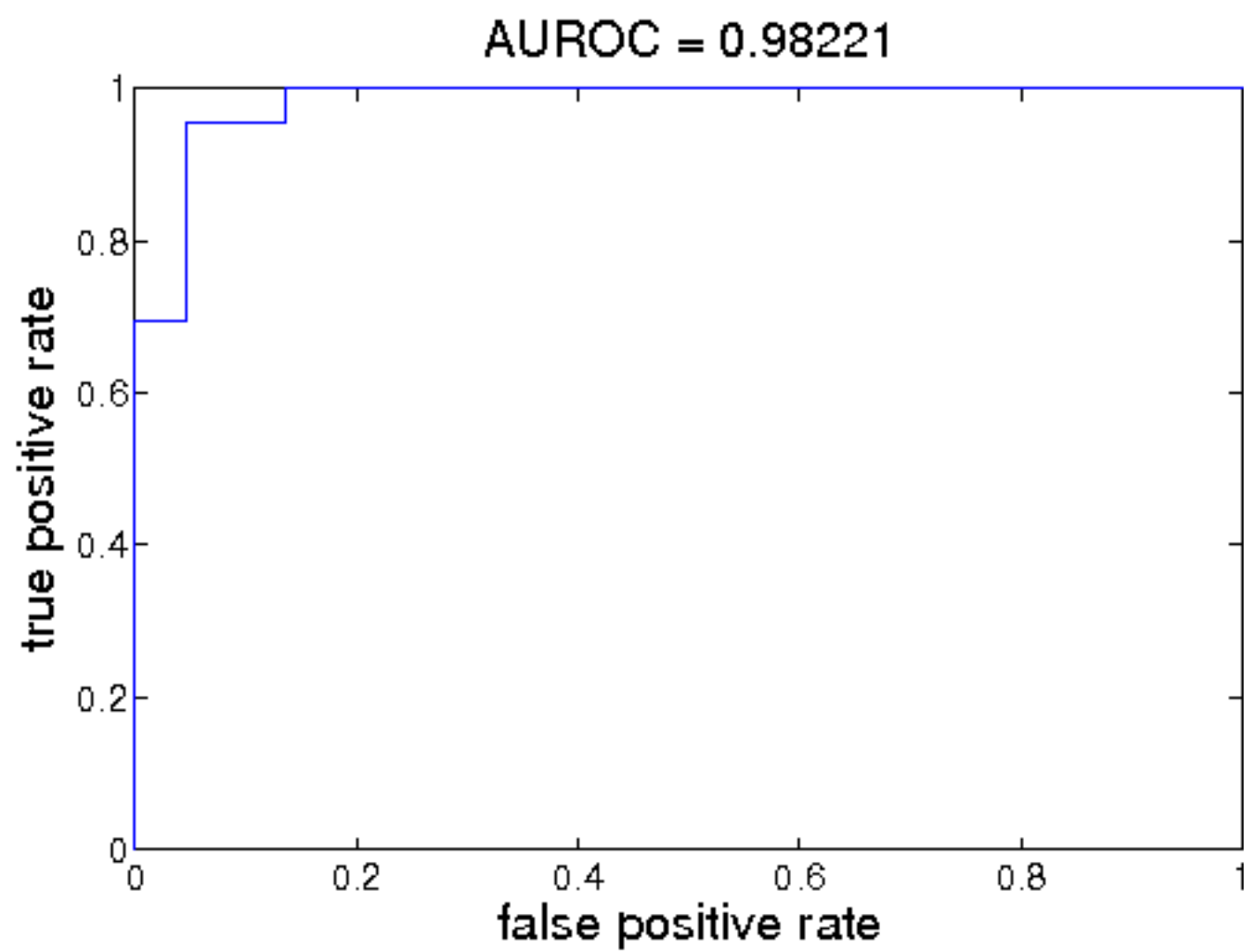

Supplement: Additional file 3 — Classification results shown as receiver operating characteristic (ROC) graphs with associated area under the curve (AUC) values for temperature adaptation, by using SVM: (A), mesophiles vs. others (B), mesothermophiles vs. others, (C) thermophiles vs. others, (D) mesophiles vs. mesothermophiles, (E) mesophiles vs. thermophiles, and (F) mesothermophiles vs. thermophiles; by using RF: (G) mesophiles vs. others, (H) mesothermophiles vs. others, (I) thermophiles vs. others, (J) mesophiles vs. mesothermophiles, (K) mesophiles vs. thermophiles, and (L) mesothermophiles vs. thermophiles. [file 1471-2148-11-26-S3.PDF]

A

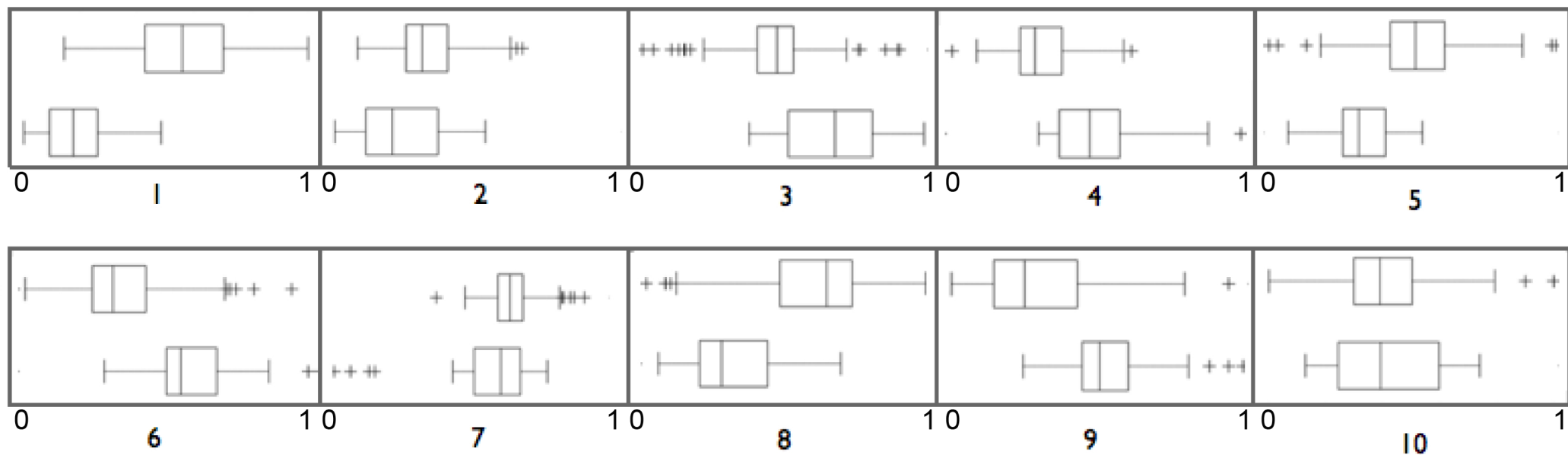

B

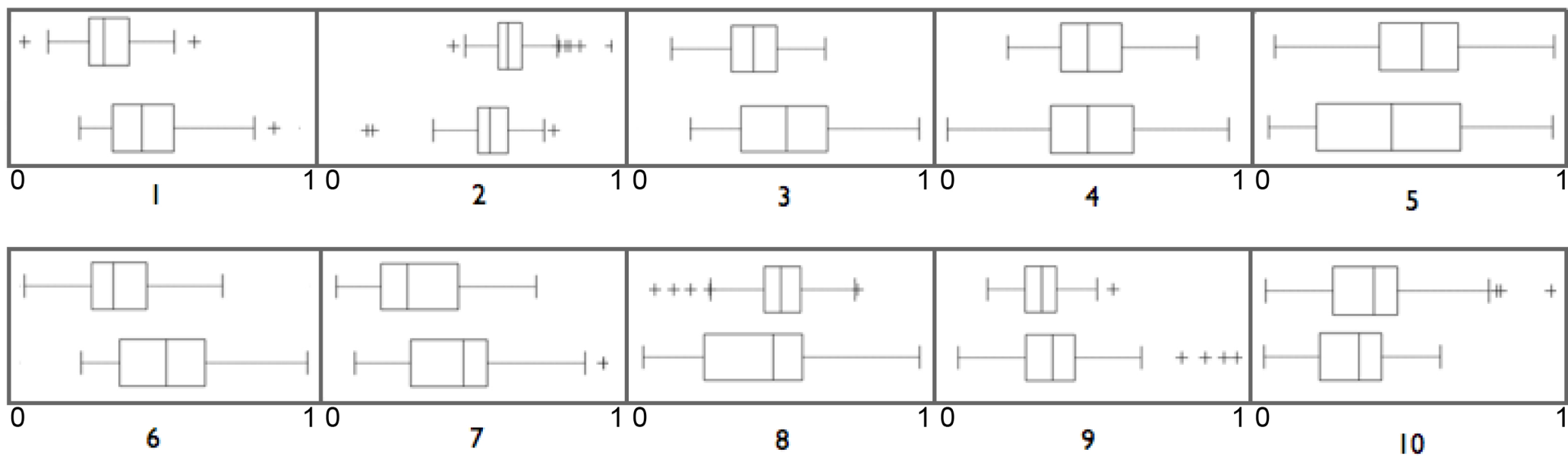

C

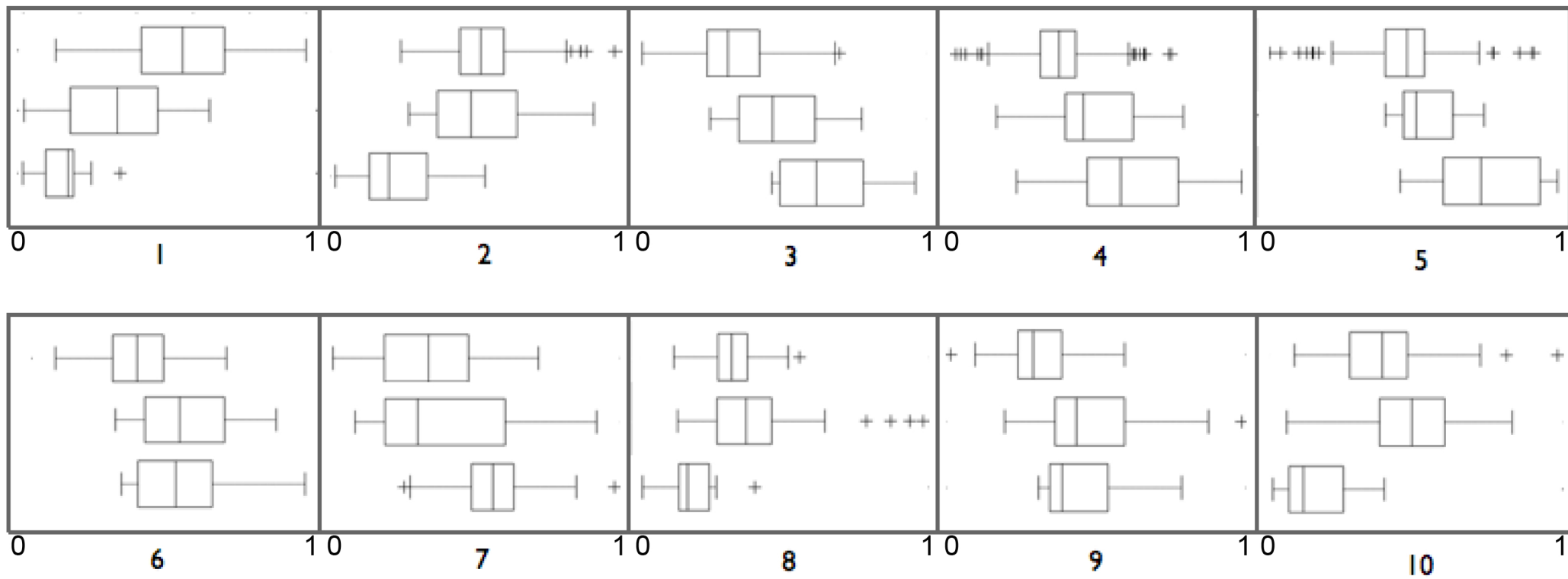

Supplement: Additional file 4 — Summary of feature selection results. (A) Ten most important features for classifications regarding domain of life revealed by the feature selection algorithm of RF. Pairs of box-and-whisker plots are shown for each feature labeled with a number: 1-Gln content, 2-Leu content, 3-normalized frequency of extended structure, 4-negative charge, 5-average protein size in a proteome, 6-Glu content, 7-charge, 8-His content, 9-ratio of charged and non-charged amino acids, 10-Cys content. Box-and-whisker plots represent bacteria and archaea from top to bottom. (B) Ten most important features for classifications regarding halophilicity revealed by the feature selection algorithm of RF. Pairs of box-and-whisker plots are shown for each feature labeled with a number: 1-negative charge, 2-charge, 3-hydrophilicity value, 4-positive charge, 5-Gln content, 6-Glu content, 7-ratio of charged and non-charged amino acids, 8-normalized frequency of beta turn, 9-Asp content, 10-Phe content. Box-and-whisker plots represent non-halophiles and halophiles from top to bottom. (C) Ten most important features for classifications regarding thermophilicity revealed by the feature selection algorithm of RF. Triplets of box-and-whisker plots are shown for each feature labeled with a number: 1-Gln content, 2-information measure for loop, 3-Glu content, 4-Val content, 5-normalized frequency of extended structure, 6-hydrophilicity value, 7-Tyr content, 8-Asp content, 9-negative charge, 10-Chou-Fasman parameter of the coil conformation. Box-and-whisker plots represent mesophiles, mesothermophiles and thermophiles from top to bottom. In all plots feature values are normalized from 0 to 1 from left to right. (+) signs represent outliers. [file 1471-2148-11-26-S4.PDF]
