# Supplementary material for: Proteome sequence features carry signatures of the environmental niche of prokaryotes
Source: BMC Evol Biol. 2011 Jan 26;11:26. doi: 10.1186/1471-2148-11-26 (PMC3045906; doi:10.1186/1471-2148-11-26)
Supplement: Additional file 7 — Final list of 42 used features in the study, together with their codes. [file 1471-2148-11-26-S7.RTF]

C1 Average non-bonded energy per atom (Oobatake-Ooi, 1977) C5 Number of hydrogen bond donors (Fauchere et al., 1988) C6 Polarity (Grantham, 1974) C7 Hydrophilicity value (Hopp-Woods, 1981) C9 Long range non-bonded energy per atom (Oobatake-Ooi, 1977) C10 Negative charge (Fauchere et al., 1988) C11 Positive charge (Fauchere et al., 1988)C13 Normalized relative frequency of bend (Isogai et al., 1980) C14 Normalized frequency of beta-turn (Chou-Fasman, 1978a) C15 Molecular weight (Fasman, 1976) C17 Normalized frequency of coil (Nagano, 1973) C21 Optimized propensity to form reverse turn (Oobatake et al., 1985)  C22 The Chou-Fasman parameter of the coil conformation (Charton-Charton, 1983) C23 Information measure for loop (Robson-Suzuki, 1976) C27 Alpha-helix indices (Geisow-Roberts, 1980) C30 Normalized frequency of turn (Crawford et al., 1973) C34 Normalized relative frequency of coil (Isogai et al., 1980) C35 Normalized frequency of left-handed alpha-helix (Maxfield-Scheraga, 1976) C37 Free energy in alpha-helical region (Munoz-Serrano, 1994) C39 Normalized frequency of extended structure (Burgess et al., 1974) C42 Information measure for pleated-sheet (Robson-Suzuki, 1976) C43 Hydropathy index (Kyte-Doolittle, 1982) C44 Consensus normalized hydrophobicity scale (Eisenberg, 1984) C49 Charge Avg Average protein size in proteomeA composition R compositionD compositionC compositionQ composition	E compositionH compositionL compositionM compositionF compositionS compositionT compositionW compositionY compositionV compositionChg Charged/Noncharged ratioDisAll Disordered/All ratio
