# Supplementary material for: Proteome sequence features carry signatures of the environmental niche of prokaryotes
Source: BMC Evol Biol. 2011 Jan 26;11:26. doi: 10.1186/1471-2148-11-26 (PMC3045906; doi:10.1186/1471-2148-11-26)
Supplement: Additional file 1 — List of 1107 species used in this study, with values of each feature. Codes used for feature names are listed in Additional file 7. [file 1471-2148-11-26-S1.PDF]

Bac=1, Arh=0	NonHal=1, Hal=1	Mez=0, Ter=1, MeTe=2	HAMAP code of the species	C1	C5	C6	C7	C9	C10	C11	C13	C14	C15	C17	C21	C22	C23	C27	C30	C34	C35	C37	C39	C42	C43	C44	C49	Avg	A	R	D	C	Q	E	H	L	M	F	S	T	W	Y	V	Chg	DisAll
1	1	0	ACIAD	-1.75787028509	0.778894756331	8.35791030324	-0.107461045506	-0.540366655562	0.107237014029	0.126507867238	0.938852438325	0.945075860534	130.11510304	0.94822600378	1.2208893504	0.968620698894	-0.870944231838	1.05043268352	0.950675168096	0.913903011744	0.839989710881	0.921597575206	0.342113076113	-0.033044588748	-0.105142021684	-0.101756503259	0.019270853209	317.690157959	0.081980685575	0.043808415744	0.051215703531	0.010899230875	0.05578969693	0.056021310497	0.026753012862	0.104923969529	0.026761488648	0.043951936183	0.061810037762	0.052553090747	0.01291284086	0.03295521633	0.065367948145	0.312413752965	0.456965342773
1	1	0	ACIBL	-1.76184680669	0.777090374501	8.40946663896	-0.019054849615	-0.539204208922	0.111366053292	0.133698220431	0.961023450698	0.95284674688	128.049700103	0.96364064274	1.25575837039	0.981085593203	-0.832783914014	1.0410283132	0.944725493148	0.91537977024	0.860013113921	0.92986443764	0.342165578432	-0.318421455841	-0.13132843671	-0.117144274468	0.022332167139	351.562264151	0.104622068223	0.062642584993	0.051644949512	0.010183706022	0.036906403606	0.05972110378	0.023493885347	0.094520053704	0.025267891201	0.039871587008	0.060783103904	0.055927402371	0.013690271664	0.026664627753	0.075777745399	0.332821440896	0.497278737047
1	1	?	ACIC1	-1.77686336191	0.754560027678	8.33625689498	-0.021999652615	-0.546249104984	0.109668723643	0.12824677127	0.9684318639	0.946105153063	125.454358181	0.974819532365	1.29843943488	0.982785851535	-0.948132619849	1.02655228983	0.92185998755	0.894847154713	0.846940342256	0.935513559788	0.346569696382	-0.335935125256	0.007096465365	-0.106589422098	0.018578047627	338.459647495	0.136505220618	0.088416109106	0.057311947121	0.009150874399	0.0272793371	0.052356776522	0.022056629373	0.099986416949	0.017332795963	0.028436855609	0.050162959793	0.058608504839	0.013719414555	0.020355904355	0.093080353339	0.320366409846	0.520106373285
0	0	1	AERPE	-1.76924544043	0.769461770856	8.28815785254	0.017851310254	-0.54861055863	0.120486162491	0.140578643412	0.952430478654	0.935078558755	128.098602898	0.971307849878	1.22842907059	0.97386584571	-1.02132511441	1.03993215889	0.922133501013	0.914181858728	0.872505702043	0.925947549589	0.349124717053	-0.197285622486	0.007920609472	-0.109062848218	0.020092480921	289.465567981	0.095698355054	0.081618007956	0.042345376976	0.008484659783	0.01643219625	0.078140785515	0.014893305486	0.110559733939	0.023424560072	0.028507575748	0.061506898	0.040319276784	0.012594680353	0.034588552723	0.093092475983	0.36511529873	0.514882789572
1	1	0	AGRT5	-1.76864859476	0.754043283762	8.35839950581	-0.031360581492	-0.542469793681	0.112506294736	0.128694515985	0.958396950365	0.946601234814	127.234090889	0.961176600899	1.26640359656	0.977003134215	-0.908191786125	1.04399365452	0.936878725253	0.915327251241	0.864535152442	0.926551247538	0.342551414931	-0.288172477523	-0.032232439024	-0.102686809872	0.016188221249	310.774845592	0.114333003122	0.06774180922	0.054836199578	0.009221122702	0.031024042328	0.057670095157	0.020790571949	0.09859981236	0.027212291127	0.041123255379	0.059707491628	0.053199753614	0.012727993333	0.022746142679	0.072766263056	0.325753749874	0.499406312281
1	0	0	ALCBS	-1.76322015799	0.774598078363	8.39976429066	-0.032163195866	-0.539754095897	0.11747585703	0.126601860535	0.956379446375	0.951517456187	128.431936075	0.957137363752	1.26097110787	0.976831345007	-0.871472758718	1.04845205844	0.941362540531	0.91831395376	0.86368573882	0.924985977955	0.342166537138	-0.29217768217	-0.124629119274	-0.114711716913	0.009126003505	329.964013086	0.10009530086	0.062065722984	0.057143211546	0.010959400241	0.045572829437	0.060332645485	0.024176151788	0.110068913706	0.027628090547	0.036566215829	0.056768632754	0.050330272292	0.014818848525	0.025205665158	0.072381641942	0.330202325399	0.488446998318
1	0	0	ALHEH	-1.76568187916	0.796427091872	8.42259956102	0.032389129598	-0.542459584604	0.125908720096	0.135699685488	0.967022554395	0.948399310167	128.024607108	0.965650968584	1.28798954233	0.98045786863	-0.901277003553	1.04707173972	0.927629161637	0.909387076357	0.872671694219	0.927268083854	0.342626063242	-0.45191543054	-0.163034225135	-0.133416255922	0.009790965392	343.790283118	0.114096224079	0.08742774818	0.058152614512	0.010166860855	0.038216267778	0.067756105584	0.025624397379	0.113010418444	0.023583927313	0.031045810761	0.043856389585	0.046850333559	0.015436899508	0.023176237874	0.075033994959	0.362620255016	0.515864570007
1	0	0	ALKMQ	-1.74975562937	0.785343608866	8.43493646247	0.013644950514	-0.538246918706	0.130025776274	0.136971095109	0.939416917689	0.938363638447	130.871716498	0.948504022909	1.24617303523	0.965566548517	-0.940226397292	1.05812611912	0.947481928943	0.937383637629	0.882125824922	0.917827387289	0.342578329171	-0.037030862127	-0.132387438017	-0.113601976331	0.006945318835	295.953425884	0.06050418948	0.040663926279	0.050969245246	0.010290889864	0.034928323715	0.079056531029	0.018750110868	0.09462080905	0.032284598991	0.041492444498	0.056963639462	0.050769203887	0.007870035506	0.036666461077	0.068884950989	0.375323127866	0.447844806753
1	1	0	ANADE	-1.7744495814	0.757471977032	8.37817153197	0.047135451211	-0.541274009388	0.11187411821	0.140204411926	0.97061336472	0.939918468499	124.934465867	0.966898350572	1.30456665514	0.978359013751	-1.04430231147	1.04504378126	0.91219198616	0.896304440487	0.865691732235	0.925392146149	0.342704741124	-0.615143936433	-0.034989793324	-0.120544862282	0.028330293716	348.9643186	0.158192925751	0.09544476889	0.050293304859	0.009581687862	0.024390644483	0.061580813351	0.019789726796	0.107632882525	0.017760455135	0.029285240992	0.041916245406	0.043604491947	0.012852648549	0.01765693586	0.08421457642	0.345182213861	0.563009127502
1	1	0	ANAMM	-1.77099324524	0.751583365284	8.30242534206	-0.057719235202	-0.545654376999	0.105020948071	0.134519031033	0.945058296336	0.951840345779	127.674707938	0.966411287404	1.24510443609	0.97963707221	-0.825756306751	1.0329349742	0.944927337273	0.92997016383	0.88186702181	0.927965565111	0.348649734172	-0.032725348398	0.030456952312	-0.098679617775	0.029498082962	359.344279661	0.089484463061	0.05887786032	0.049039455309	0.021290673429	0.027443554801	0.055981492762	0.023101561383	0.093992548408	0.028994942399	0.040201502574	0.079192601223	0.045333230795	0.008987097465	0.029657966073	0.091271142656	0.321756034179	0.47795418149
1	1	0	ANAVT	-1.75674976679	0.793798662083	8.36757119436	-0.081208398924	[illegible]	[illegible]	[illegible]	[illegible]	[illegible]	[illegible]	[illegible]	[illegible]	[illegible]	[illegible]	[illegible]	[illegible]	[illegible]	[illegible]	[illegible]	[illegible]	[illegible]	[illegible]	[illegible]	[illegible]	[illegible]	[illegible]	[illegible]	[illegible]	[illegible]	[illegible]	[illegible]	[illegible]	[illegible]	[illegible]	[illegible]	[illegible]	[illegible]	[illegible]	[illegible]	[illegible]	[illegible]	[illegible]
1	0	1	AQUAE	-1.74231573539	0.797290389168	8.43123572485	0.097127589766	[illegible]	[illegible]	[illegible]	[illegible]	[illegible]	[illegible]	[illegible]	[illegible]	[illegible]	[illegible]	[illegible]	[illegible]	[illegible]	[illegible]	[illegible]	[illegible]	[illegible]	[illegible]	[illegible]	[illegible]	[illegible]	[illegible]	[illegible]	[illegible]	[illegible]	[illegible]	[illegible]	[illegible]	[illegible]	[illegible]	[illegible]	[illegible]	[illegible]	[illegible]	[illegible]	[illegible]	[illegible]	[illegible]
0	0	1	ARCFU	-1.75543129464	0.773599592904	8.37935453091	0.065810822102	[illegible]	[illegible]	[illegible]	[illegible]	[illegible]	[illegible]	[illegible]	[illegible]	[illegible]	[illegible]	[illegible]	[illegible]	[illegible]	[illegible]	[illegible]	[illegible]	[illegible]	[illegible]	[illegible]	[illegible]	[illegible]	[illegible]	[illegible]	[illegible]	[illegible]	[illegible]	[illegible]	[illegible]	[illegible]	[illegible]	[illegible]	[illegible]	[illegible]	[illegible]	[illegible]	[illegible]	[illegible]	[illegible]
1	1	0	ARTAT	-1.77420932669	0.72321124462	8.36802495944	-0.048544577742	[illegible]	[illegible]	[illegible]	[illegible]	[illegible]	[illegible]	[illegible]	[illegible]	[illegible]	[illegible]	[illegible]	[illegible]	[illegible]	[illegible]	[illegible]	[illegible]	[illegible]	[illegible]	[illegible]	[illegible]	[illegible]	[illegible]	[illegible]	[illegible]	[illegible]	[illegible]	[illegible]	[illegible]	[illegible]	[illegible]	[illegible]	[illegible]	[illegible]	[illegible]	[illegible]	[illegible]	[illegible]	[illegible]
1	0	0	AZOSE	-1.76701089979	0.793725899883	8.38691139688	0.011319123663	[illegible]	[illegible]	[illegible]	[illegible]	[illegible]	[illegible]	[illegible]	[illegible]	[illegible]	[illegible]	[illegible]	[illegible]	[illegible]	[illegible]	[illegible]	[illegible]	[illegible]	[illegible]	[illegible]	[illegible]	[illegible]	[illegible]	[illegible]	[illegible]	[illegible]	[illegible]	[illegible]	[illegible]	[illegible]	[illegible]	[illegible]	[illegible]	[illegible]	[illegible]	[illegible]	[illegible]	[illegible]	[illegible]
1	1	0	BACAN	-1.75134421767	0.780451403887	8.38487766455	-0.036748292442	[illegible]	[illegible]	[illegible]	[illegible]	[illegible]	[illegible]	[illegible]	[illegible]	[illegible]	[illegible]	[illegible]	[illegible]	[illegible]	[illegible]	[illegible]	[illegible]	[illegible]	[illegible]	[illegible]	[illegible]	[illegible]	[illegible]	[illegible]	[illegible]	[illegible]	[illegible]	[illegible]	[illegible]	[illegible]	[illegible]	[illegible]	[illegible]	[illegible]	[illegible]	[illegible]	[illegible]	[illegible]	[illegible]
1	1	0	BACCZ	-1.7517955648	0.777931672698	8.38422246084	-0.041952543693	[illegible]	[illegible]	[illegible]	[illegible]	[illegible]	[illegible]	[illegible]	[illegible]	[illegible]	[illegible]	[illegible]	[illegible]	[illegible]	[illegible]	[illegible]	[illegible]	[illegible]	[illegible]	[illegible]	[illegible]	[illegible]	[illegible]	[illegible]	[illegible]	[illegible]	[illegible]	[illegible]	[illegible]	[illegible]	[illegible]	[illegible]	[illegible]	[illegible]	[illegible]	[illegible]	[illegible]	[illegible]	[illegible]
1	1	0	BACFR	-1.75042872662	0.808978780725	8.38857240433	-0.038039892952	[illegible]	[illegible]	[illegible]	[illegible]	[illegible]	[illegible]	[illegible]	[illegible]	[illegible]	[illegible]	[illegible]	[illegible]	[illegible]	[illegible]	[illegible]	[illegible]	[illegible]	[illegible]	[illegible]	[illegible]	[illegible]	[illegible]	[illegible]	[illegible]	[illegible]	[illegible]	[illegible]	[illegible]	[illegible]	[illegible]	[illegible]	[illegible]	[illegible]	[illegible]	[illegible]	[illegible]	[illegible]	[illegible]
