# Supplementary material for: Proteome sequence features carry signatures of the environmental niche of prokaryotes
Source: BMC Evol Biol. 2011 Jan 26;11:26. doi: 10.1186/1471-2148-11-26 (PMC3045906; doi:10.1186/1471-2148-11-26)
Supplement: Additional file 5 — Histograms showing shapes of distributions of features' values within six representative proteomes: a mesophilic, thermophilic and halophilic bacterium, and a mesophilic, thermophilic and halophilic Archaeon. [file 1471-2148-11-26-S5.PDF]

Proteomes chosen as representatives:

<u>Bacteria (all three are from Firmicutes)</u>

Bacillus subtilis (non-thermophile, non-halophile), TaxID = 224308
Oceanobacillus iheyensis (non-thermophile, **halophile**), TaxID = 221109
Geobacillus thermodenitrificans NG80-2 (**thermophile**, non-halophile), TaxID= 420246

<u>Archaea (all three are from Euryarchaeota)</u>

Methanocorpusculum labreanum Z (non-themophile, non-halophile) , TaxID = 410358
Halobacterium sp. NRC-1 (non-thermophile, **halophile**), TaxID = 64091
Thermococcus kodakarensis KOD1 (**thermophile**, non-halophile), TaxID= 69014

Bacteria (Ha=halophile, Me=mesophile, Th=thermophile)

Archaea (H

#C7 Hydrophilicity value (Hopp-Woods, 1981)

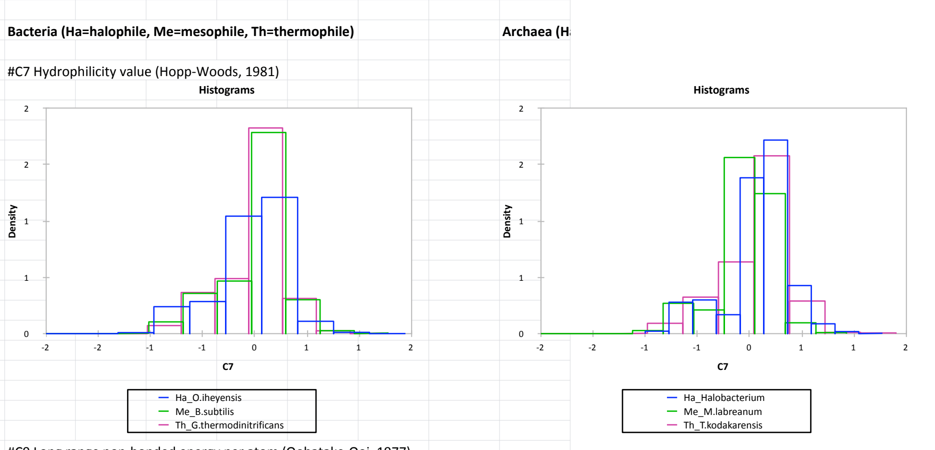

#C9 Long range non-bonded energy per atom (Oobatake-Ooi, 1977)

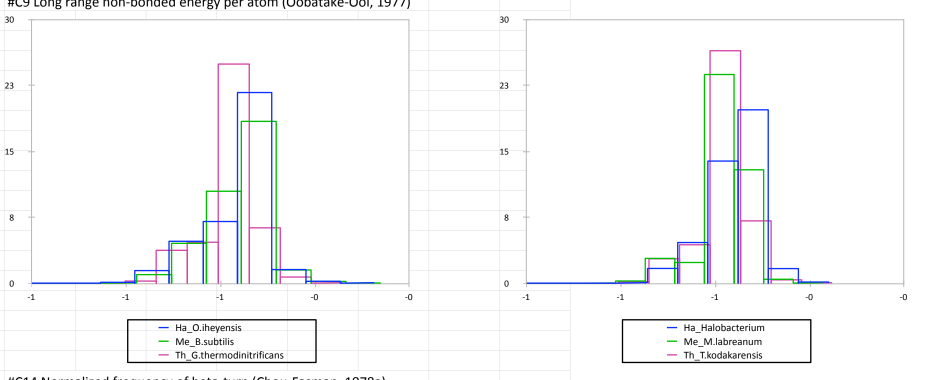

#C14 Normalized frequency of beta-turn (Chou-Fasman, 1978a)

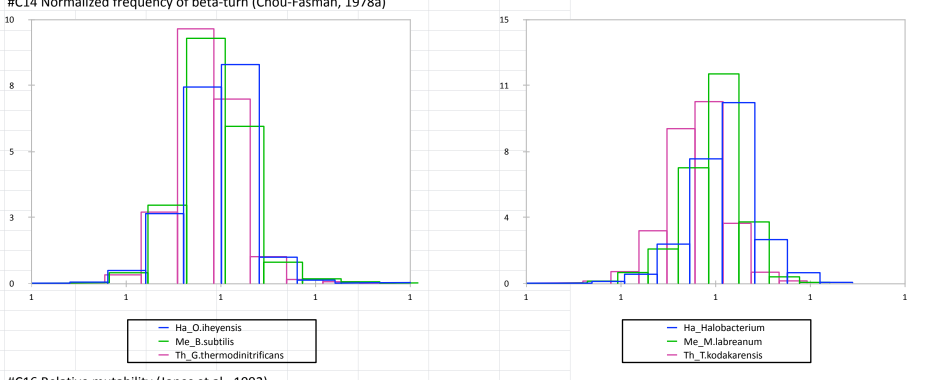

#C16 Relative mutability (Jones et al., 1992)

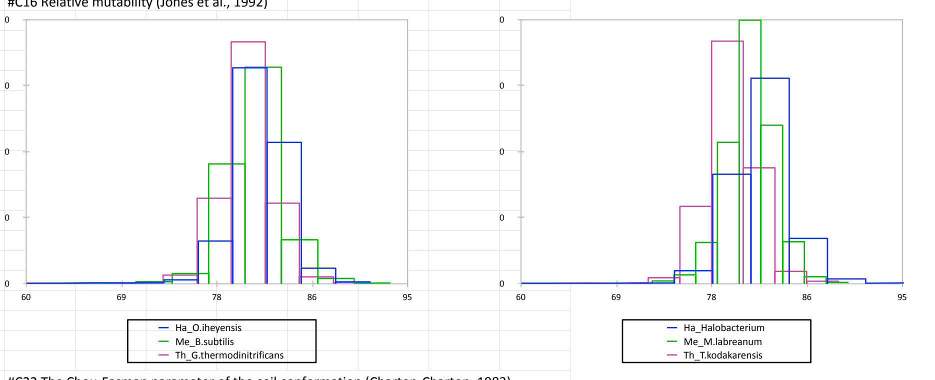

#C22 The Chou-Fasman parameter of the coil conformation (Charton-Charton, 1983)

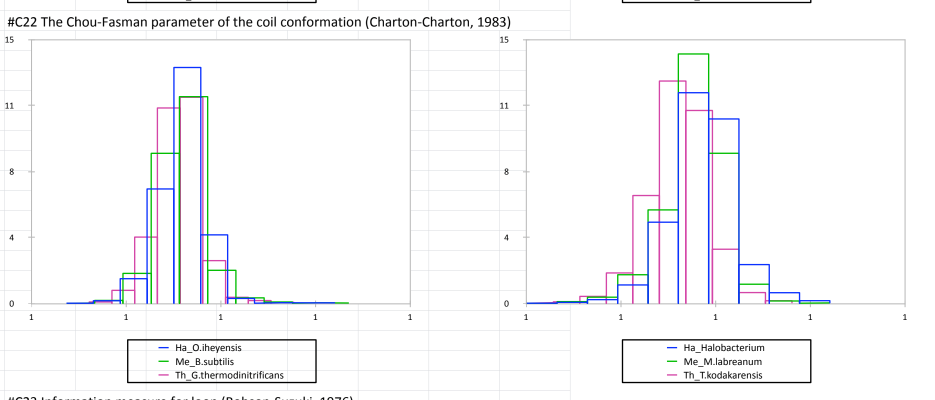

#C23 Information measure for loop (Robson-Suzuki, 1976)

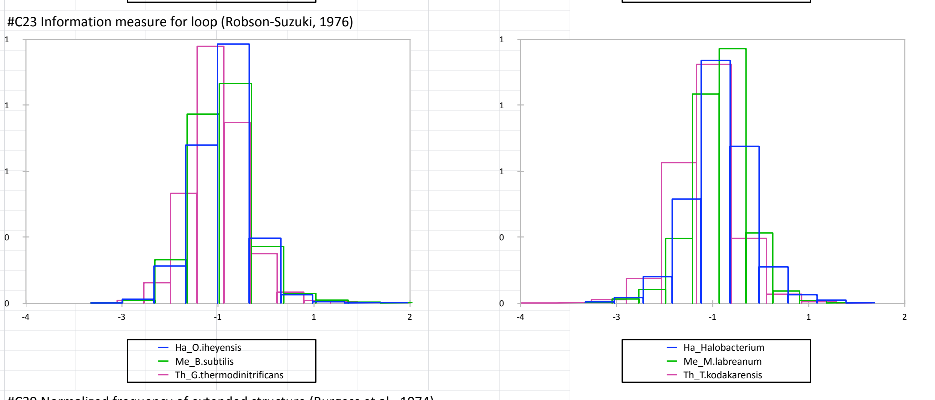

#C39 Normalized frequency of extended structure (Burgess et al., 1974)

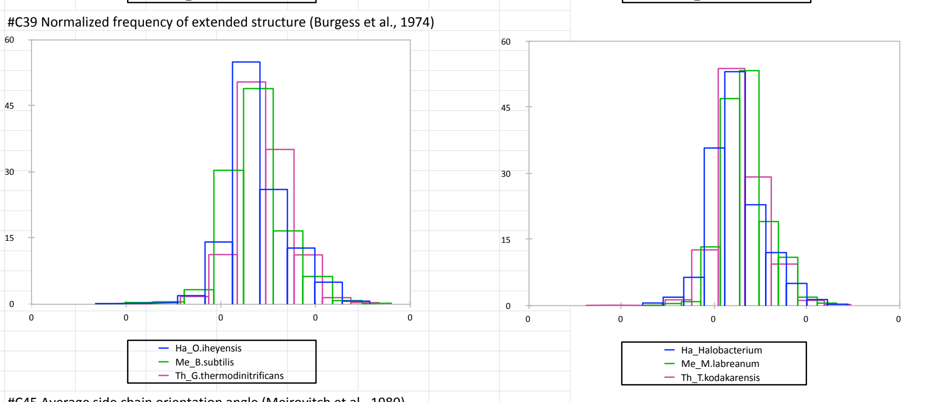

#C45 Average side chain orientation angle (Meirovitch et al., 1980)

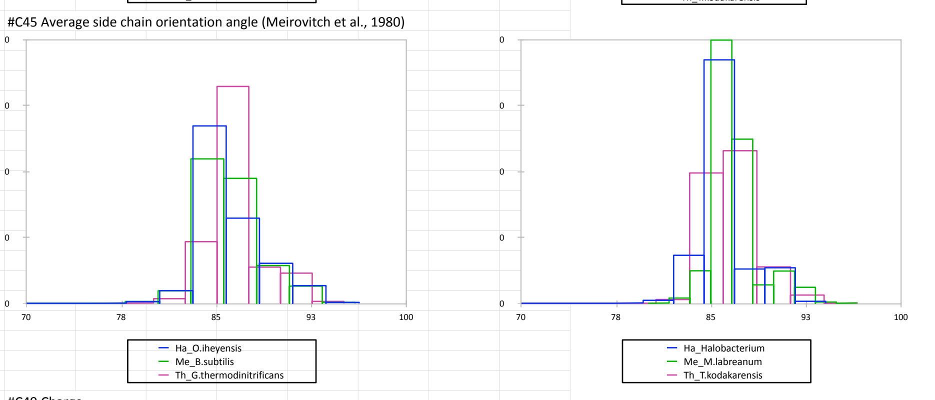

#C49 Charge

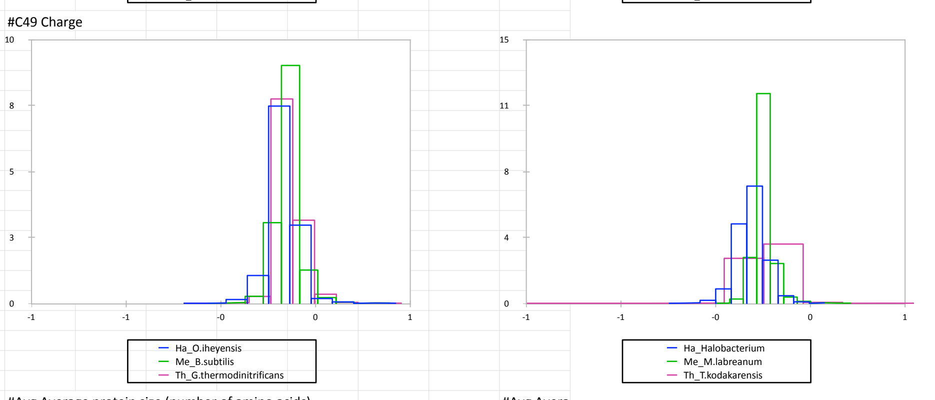

#Avg Average protein size (number of amino acids)

#Avg Avera

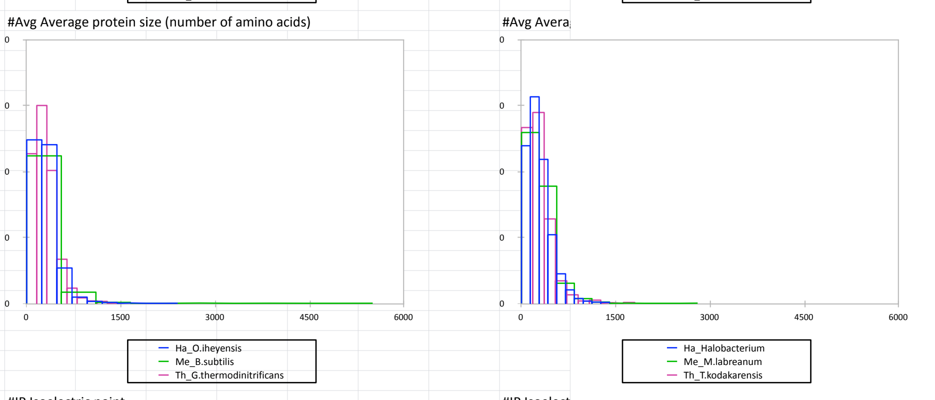

#IP Isoelectric point

#IP Isoelect

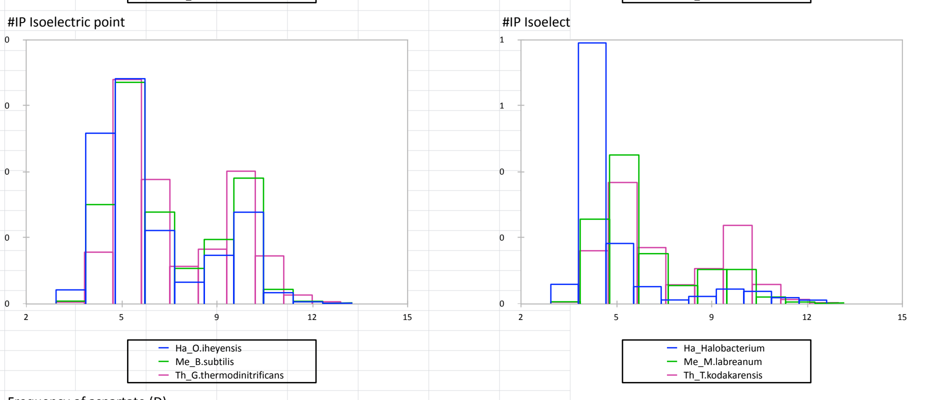

Frequency of aspartate (D)

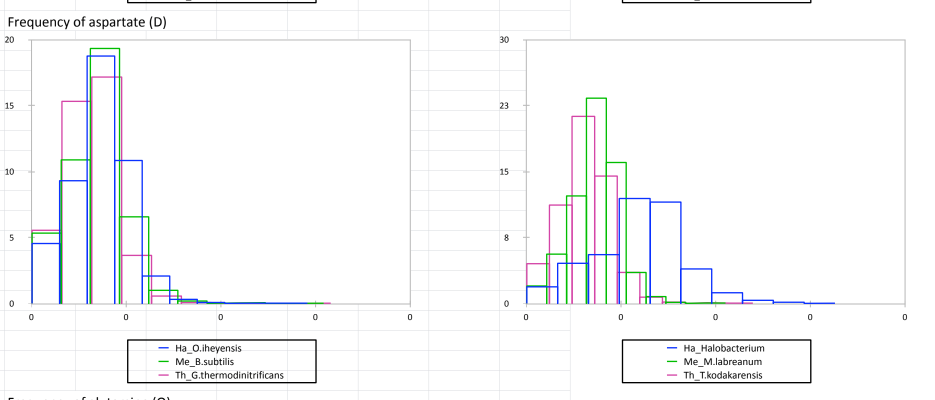

Frequency of glutamine (Q)

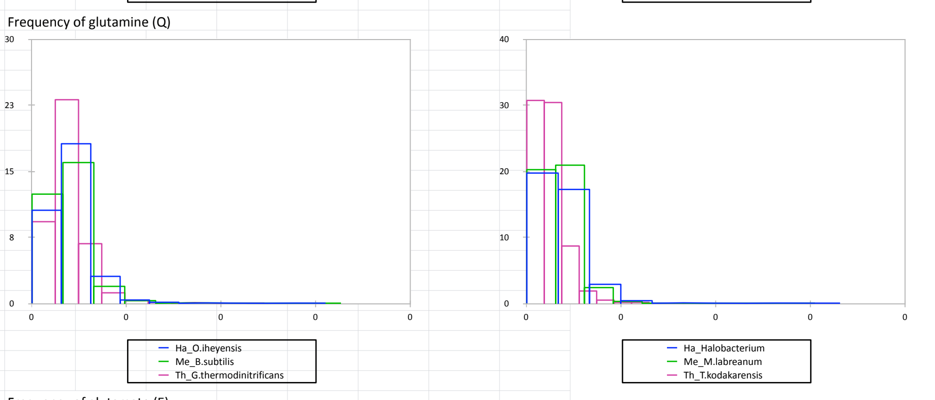

Frequency of glutamate (E)

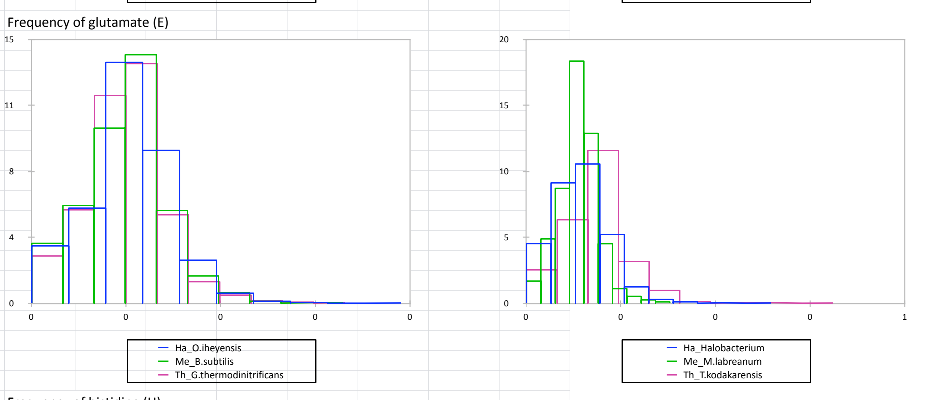

Frequency of histidine (H)

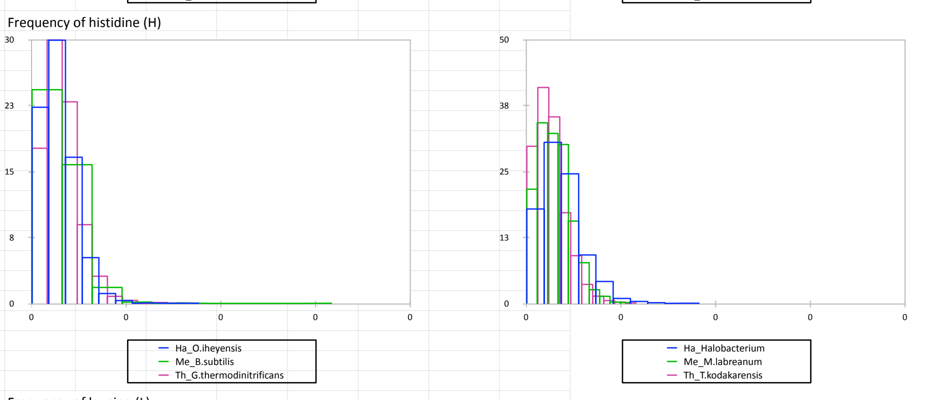

Frequency of leucine (L)

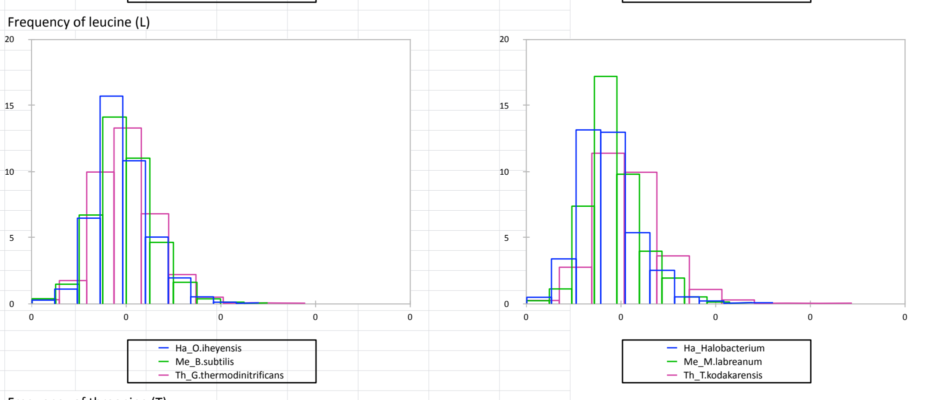

Frequency of threonine (T)

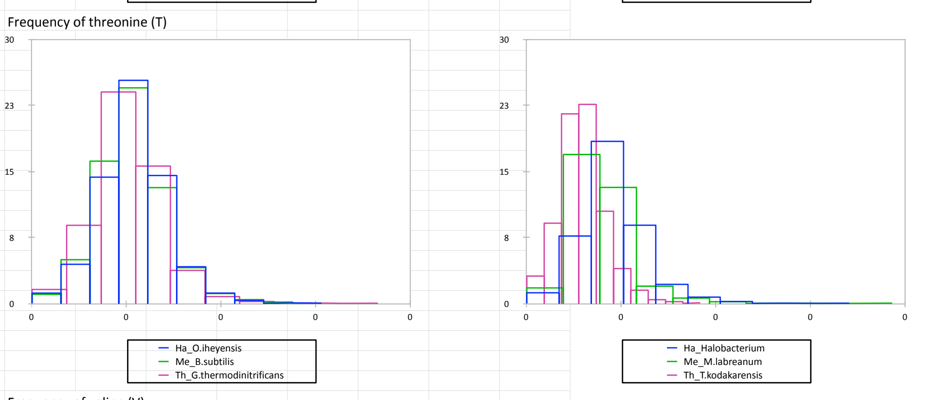

Frequency of valine (V)

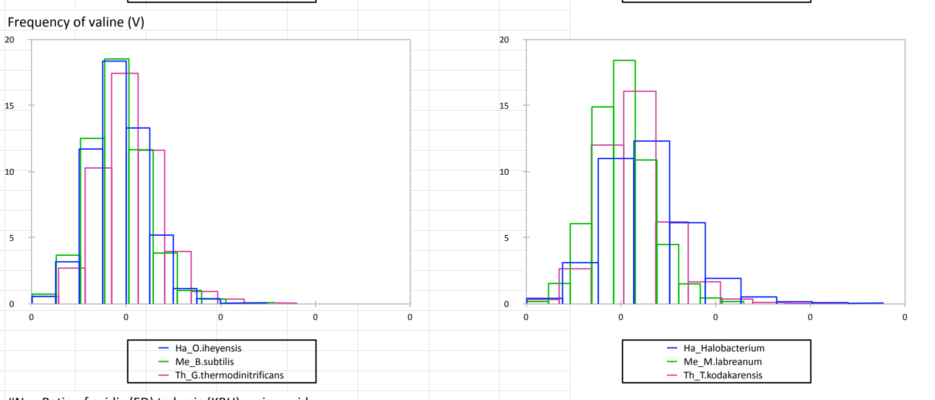

#Neg Ratio of acidic (ED) to basic (KRH) amino acids

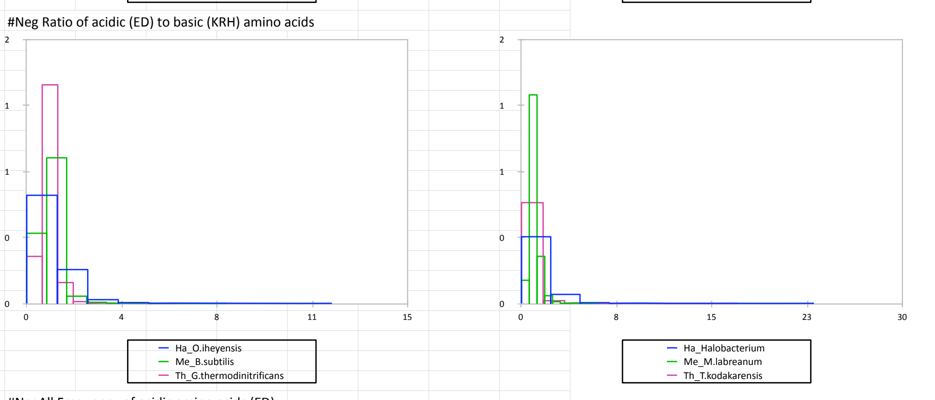

#NegAll Frequency of acidic amino acids (ED)

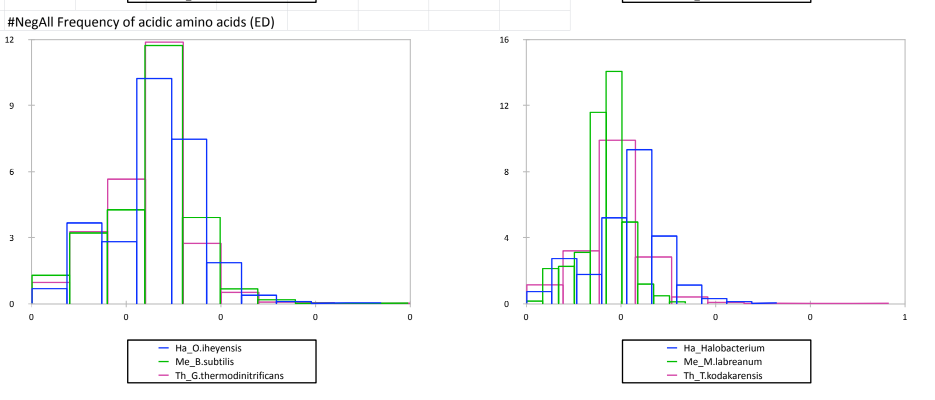

0	0			0	0			0	0
0	1			0	0			0	0
0	1			0	0			0	0
0	0			0	0			0	0
0	2			0	0			0	0
0	2			0	0			0	0
0	0			0	0			0	0
0	6			0	1			0	0
0	6			0	1			0	0
0	0			0	0			0	0
0	36			0	13			0	1
0	36			0	13			0	1
0	0			0	0			0	0
0	53			0	47			0	13
0	53			0	47			0	13
0	0			0	0			0	0
0	23			0	53			0	54
0	23			0	53			0	54
0	0			0	0			0	0
0	12			0	19			0	29
0	12			0	19			0	29
0	0			0	0			0	0
0	5			0	11			0	9
0	5			0	11			0	9
0	0			0	0			0	0
0	1			0	2			0	1
0	1			0	2			0	1
0	0			0	0			0	0
0	0			0	1			0	0
0	0			0	1			0	0
0	0			0	0			0	0

-1	0	-2	0	-2	0	-1	0	-1	0	-1	0	1	0	1	0	1	0	60	0	70	0	60	0	1	0	1	0	1	0	120	0	120	0	120	0	1	0	1	0	1	0
-1	0	-2	0	-2	0	-1	0	-1	0	-1	0	1	0	1	0	1	0	60	0	70	0	60	0	1	0	1	0	1	0	120	0	120	0	120	0	1	0	1	0	1	0
-1	0	-2	0	-2	0	-1	0	-1	0	-1	0	1	0	1	0	1	0	64	0	72	0	63	0	1	0	1	0	1	0	124	0	124	0	126	0	1	0	1	0	1	0
-1	0	-2	0	-2	0	-1	0	-1	0	-1	0	1	0	1	0	1	0	64	0	72	0	63	0	1	0	1	0	1	0	124	0	124	0	126	0	1	0	1	0	1	0
-1	0	-2	0	-2	0	-1	0	-1	0	-1	0	1	0	1	0	1	0	64	0	72	0	63	0	1	0	1	0	1	0	124	0	124	0	126	0	1	0	1	0	1	0
-1	0	-1	0	-1	0	-1	0	-1	0	-1	0	1	0	1	0	1	0	67	0	74	0	66	0	1	0	1	0	1	0	128	0	128	0	132	0	1	0	1	0	1	0
-1	0	-1	0	-1	0	-1	0	-1	0	-1	0	1	0	1	0	1	0	67	0	74	0	66	0	1	0	1	0	1	0	128	0	128	0	132	0	1	0	1	0	1	0
-1	0	-1	0	-1	0	-1	0	-1	0	-1	0	1	0	1	0	1	0	67	0	74	0	66	0	1	0	1	0	1	1	128	0	128	0	132	0	1	0	1	0	1	0
-0	0	-1	0	-1	0	-1	0	-1	0	-1	0	1	0	1	0	1	0	71	0	76	0	69	0	1	0	1	0	1	1	132	0	132	0	138	0	1	0	1	0	1	0
-0	0	-1	0	-1	0	-1	0	-1	0	-1	0	1	0	1	0	1	0	71	0	76	0	69	0	1	0	1	0	1	0	132	0	132	0	138	0	1	0	1	0	1	0
-0	0	-1	0	-1	0	-1	0	-1	0	-1	0	1	1	1	1	1	1	71	0	76	0	69	0	1	2	1	1	1	3	132	0	132	0	138	0	1	1	1	1	1	1
-0	0	-1	0	-1	0	-1	0	-1	0	-1	0	1	1	1	1	1	1	74	0	78	0	72	0	1	2	1	1	1	3	136	0	136	0	144	0	1	1	1	1	1	1
-0	0	-1	0	-1	0	-1	0	-1	0	-1	0	1	0	1	0	1	0	74	0	78	0	72	0	1	0	1	0	1	0	136	0	136	0	144	0	1	0	1	0	1	0
-0	1	-1	0	-1	0	-1	2	-1	3	-1	3	1	2	1	2	1	3	74	0	78	0	72	0	1	11	1	5	1	10	136	0	136	0	144	0	1	4	1	4	1	5
0	1	-1	0	-0	0	-1	2	-1	3	-1	3	1	2	1	2	1	3	78	0	80	0	75	0	1	11	1	5	1	10	141	0	141	0	150	0	1	4	1	4	1	5
0	0	-1	0	-0	0	-1	0	-1	0	-1	0	1	0	1	0	1	0	78	0	80	0	75	0	1	0	1	0	1	0	141	0	141	0	150	0	1	0	1	0	1	0
0	2	-1	0	-0	1	-1	5	-1	2	-1	4	1	7	1	7	1	9	78	0	80	0	75	0	1	13	1	15	1	14	141	0	141	0	150	0	1	12	1	14	1	16
0	2	-0	0	0	1	-1	5	-1	2	-1	4	1	7	1	7	1	9	81	0	82	0	77	0	1	13	1	15	1	14	145	0	145	0	155	0	1	12	1	14	1	16
0	0	-0	0	0	0	-1	0	-1	0	-1	0	1	0	1	0	1	0	81	0	82	0	77	0	1	0	1	0	1	0	145	0	145	0	155	0	1	0	1	0	1	0
0	0	-0	2	0	2	-1	14	-1	24	-1	26	1	10	1	12	1	10	81	0	82	0	77	0	1	3	1	11	1	5	145	0	145	0	155	0	1	14	1	14	1	11
1	0	0	2	0	2	-1	14	-1	24	-1	26	1	10	1	12	1	10	85	0	84	0	80	0	1	3	1	11	1	5	149	0	149	0	161	0	1	14	1	14	1	11
1	0	0	0	0	0	-1	0	-1	0	-1	0	1	0	1	0	1	0	85	0	84	0	80	0	1	0	1	0	1	0	149	0	149	0	161	0	1	0	1	0	1	0
1	0	0	1	0	0	-1	20	-1	13	-1	7	1	2	1	3	1	3	85	0	84	0	80	0	1	0	1	2	1	1	149	0	149	0	161	0	1	3	1	2	1	1
1	0	0	1	1	0	-1	20	-1	13	-1	7	1	2	1	3	1	3	88	0	86	0	83	0	1	0	1	2	1	1	153	0	153	0	167	0	1	3	1	2	1	1
1	0	0	0	1	0	-1	0	-1	0	-1	0	1	0	1	0	1	0	88	0	86	0	83	0	1	0	1	0	1	0	153	0	153	0	167	0	1	0	1	0	1	0
1	0	0	0	1	0	-1	2	-1	0	-1	0	1	1	1	0	1	1	88	0	86	0	83	0	1	0	1	0	1	0	153	0	153	0	167	0	1	0	1	0	1	0
1	0	1	0	1	0	-0	2	-0	0	-0	0	1	1	1	0	1	1	92	0	88	0	86	0	1	0	1	0	1	0	157	0	157	0	173	0	1	0	1	0	1	0
1	0	1	0	1	0	-0	0	-0	0	-0	0	1	0	1	0	1	0	92	0	88	0	86	0	1	0	1	0	1	0	157	0	157	0	173	0	1	0	1	0	1	0
1	0	1	0	1	0	-0	0	-0	0	-0	0	1	0	1	0	1	0	92	0	88	0	86	0	1	0	1	0	1	0	157	0	157	0	173	0	1	0	1	0	1	0
1	0	1	0	1	0	-0	0	-0	0	-0	0	1	0	1	0	1	0	95	0	90	0	89	0	1	0	1	0	1	0	161	0	161	0	179	0	1	0	1	0	1	0
1	0	1	0	1	0	-0	0	-0	0	-0	0	1	0	1	0	1	0	95	0	90	0	89	0	1	0	1	0	1	0	161	0	161	0	179	0	1	0	1	0	1	0

110	0	120	0	120	0	0	0	1	0	0	0	1	0	1	0	1	0	-3	0	-3	0	-4	0	0	0	0	0	0	0	70	0	80	0	70	0	-0	0	-0	0	-1	0
110	0	120	0	120	0	0	0	1	0	0	0	1	0	1	0	1	0	-3	0	-3	0	-4	0	0	1	0	0	0	0	70	0	80	0	70	0	-0	0	-0	0	-1	0
114	0	123	0	125	0	0	0	1	0	0	0	1	0	1	0	1	0	-3	0	-3	0	-3	0	0	1	0	0	0	0	72	0	82	0	73	0	-0	0	-0	0	-1	0
114	0	123	0	125	0	0	0	1	0	0	0	1	0	1	0	1	0	-3	0	-3	0	-3	0	0	0	0	0	0	0	72	0	82	0	73	0	-0	0	-0	0	-1	0
114	0	123	0	125	0	0	0	1	0	0	0	1	0	1	0	1	0	-3	0	-3	0	-3	0	0	2	0	0	0	0	72	0	82	0	73	0	-0	0	-0	0	-1	0
118	0	126	0	130	0	0	0	1	0	0	0	1	0	1	0	1	0	-2	0	-2	0	-3	0	0	2	0	0	0	0	75	0	83	0	75	0	-0	0	-0	0	-1	0
118	0	126	0	130	0	0	0	1	0	0	0	1	0	1	0	1	0	-2	0	-2	0	-3	0	0	0	0	0	0	0	75	0	83	0	75	0	-0	0	-0	0	-1	0
118	0	126	0	130	0	0	0	1	0	0	0	1	0	1	0	1	0	-2	0	-2	0	-3	0	0	6	0	1	0	0	75	0	83	0	75	0	-0	0	-0	3	-1	0
122	0	130	0	135	0	1	0	1	0	1	0	1	0	1	0	1	0	-2	0	-2	0	-2	0	0	6	0	1	0	0	77	0	85	0	78	0	-0	0	-0	3	-0	0
122	0	130	0	135	0	1	0	1	0	1	0	1	0	1	0	1	0	-2	0	-2	0	-2	0	0	0	0	0	0	0	77	0	85	0	78	0	-0	0	-0	0	-0	0
122	0	130	0	135	0	1	0	1	0	1	0	1	1	1	2	1	2	-2	0	-2	0	-2	0	0	36	0	13	0	1	77	0	85	0	78	0	-0	1	-0	12	-0	0
126	0	133	0	140	0	1	0	1	0	1	0	1	1	1	2	1	2	-1	0	-1	0	-2	0	0	36	0	13	0	1	80	0	87	0	81	0	-0	1	0	12	-0	0
126	0	133	0	140	0	1	0	1	0	1	0	1	0	1	0	1	0	-1	0	-1	0	-2	0	0	0	0	0	0	0	80	0	87	0	81	0	-0	0	0	0	-0	0
126	0	133	0	140	0	1	0	1	1	1	0	1	5	1	5	1	6	-1	1	-1	1	-2	1	0	53	0	47	0	13	80	0	87	0	81	0	-0	5	0	2	-0	0
130	0	136	0	146	0	1	0	1	1	1	0	1	5	1	5	1	6	-1	1	-1	1	-1	1	0	53	0	47	0	13	82	0	88	0	83	0	-0	5	0	2	-0	0
130	0	136	0	146	0	1	0	1	0	1	0	1	0	1	0	1	0	-1	0	-1	0	-1	0	0	0	0	0	0	0	82	0	88	0	83	0	-0	0	0	0	-0	0
130	0	136	0	146	0	1	0	1	1	1	0	1	12	1	14	1	13	-1	1	-1	1	-1	1	0	23	0	53	0	54	82	0	88	0	83	0	-0	7	0	0	-0	3
134	0	139	0	151	0	1	0	1	1	1	0	1	12	1	14	1	13	-0	1	-0	1	-1	1	0	23	0	53	0	54	84	0	90	0	86	0	-0	7	0	0	0	3
134	0	139	0	151	0	1	0	1	0	1	0	1	0	1	0	1	0	-0	0	-0	0	-1	0	0	0	0	0	0	0	84	0	90	0	86	0	-0	0	0	0	0	0
134	0	139	0	151	0	1	1	1	4	1	1	1	10	1	9	1	11	-0	0	-0	0	-1	0	0	12	0	19	0	29	84	0	90	0	86	0	-0	2	0	0	0	3
138	0	142	0	156	0	1	1	1	4	1	1	1	10	1	9	1	11	0	0	-0	0	-0	0	0	12	0	19	0	29	87	0	92	0	89	0	0	2	0	0	0	3
138	0	142	0	156	0	1	0	1	0	1	0	1	0	1	0	1	0	0	0	-0	0	-0	0	0	0	0	0	0	0	87	0	92	0	89	0	0	0	0	0	0	0
138	0	142	0	156	0	1	3	1	5	1	4	1	2	1	1	1	3	0	0	-0	0	-0	0	0	5	0	11	0	9	87	0	92	0	89	0	0	0	0	0	0	0
142	0	146	0	161	0	2	3	1	5	1	4	1	2	1	1	1	3	1	0	0	0	0	0	0	5	0	11	0	9	89	0	93	0	91	0	0	0	0	0	0	0
142	0	146	0	161	0	2	0	1	0	1	0	1	0	1	0	1	0	1	0	0	0	0	0	0	0	0	0	0	0	89	0	93	0	91	0	0	0	0	0	0	0
142	0	146	0	161	0	2	0	1	1	1	0	1	1	1	0	1	1	1	0	0	0	0	0	0	1	0	2	0	1	89	0	93	0	91	0	0	0	0	0	0	0
146	0	149	0	166	0	2	0	1	1	2	0	1	1	1	0	1	1	1	0	1	0	1	0	0	1	0	2	0	1	92	0	95	0	94	0	0	0	0	0	1	0
146	0	149	0	166	0	2	0	1	0	2	0	1	0	1	0	1	0	1	0	1	0	1	0	0	0	0	0	0	0	92	0	95	0	94	0	0	0	0	0	1	0
146	0	149	0	166	0	2	0	1	0	2	0	1	0	1	0	1	0	1	0	1	0	1	0	0	0	0	1	0	0	92	0	95	0	94	0	0	0	0	0	1	0
150	0	152	0	171	0	2	0	2	0	2	0	1	0	1	0	1	0	2	0	1	0	1	0	0	0	0	1	0	0	94	0	97	0	97	0	0	0	0	0	1	0
150	0	152	0	171	0	2	0	2	0	2	0	1	0	1	0	1	0	2	0	1	0	1	0	0	0	0	0	0	0	94	0	97	0	97	0	0	0	0	0	1	0

0	0	0	0	0	0	3	0	3	0	3	0	0	0	0	0	0	0	0	0	0	0	0	0	0	0	0	0	0	0	0	0	0	0	0	0	0	0	0	0	0	0
0	0	0	0	0	0	3	0	3	0	3	0	0	2	0	2	0	5	0	20	0	20	0	31	0	5	0	2	0	3	0	1	0	0	0	1	0	18	0	22	0	30
140	0	280	0	180	0	4	0	4	0	4	0	0	2	0	2	0	5	0	20	0	20	0	31	0	5	0	2	0	3	0	1	0	0	0	1	0	18	0	22	0	30
140	0	280	0	180	0	4	0	4	0	4	0	0	0	0	0	0	0	0	0	0	0	0	0	0	0	0	0	0	0	0	0	0	0	0	0	0	0	0	0	0	0
140	0	280	0	180	0	4	1	4	0	4	0	0	5	0	6	0	11	0	17	0	21	0	30	0	9	0	5	0	6	0	5	0	2	0	5	0	30	0	34	0	41
280	0	560	0	360	0	5	1	5	0	5	0	0	5	0	6	0	11	0	17	0	21	0	30	0	9	0	5	0	6	0	5	0	2	0	5	0	30	0	34	0	41
280	0	560	0	360	0	5	0	5	0	5	0	0	0	0	0	0	0	0	0	0	0	0	0	0	0	0	0	0	0	0	0	0	0	0	0	0	0	0	0	0	0
280	0	560	0	360	0	5	0	5	0	5	0	0	6	0	12	0	21	0	3	0	2	0	9	0	11	0	9	0	12	0	12	0	6	0	14	0	25	0	32	0	35
420	0	840	0	540	0	6	0	6	0	6	0	0	6	0	12	0	21	0	3	0	2	0	9	0	11	0	9	0	12	0	12	0	6	0	14	0	25	0	32	0	35
420	0	840	0	540	0	6	0	6	0	6	0	0	0	0	0	0	0	0	0	0	0	0	0	0	0	0	0	0	0	0	0	0	0	0	0	0	0	0	0	0	0
420	0	840	0	540	0	6	0	6	0	6	0	0	12	0	23	0	14	0	0	0	0	0	2	0	5	0	18	0	3	0	14	0	14	0	15	0	9	0	30	0	17
560	0	1120	0	720	0	7	0	7	0	7	0	0	12	0	23	0	14	0	0	0	0	0	2	0	5	0	18	0	3	0	14	0	14	0	15	0	9	0	30	0	17
560	0	1120	0	720	0	7	0	7	0	7	0	0	0	0	0	0	0	0	0	0	0	0	0	0	0	0	0	0	0	0	0	0	0	0	0	0	0	0	0	0	0
560	0	1120	0	720	0	7	0	7	0	7	0	0	12	0	16	0	4	0	0	0	0	0	0	0	1	0	13	0	1	0	5	0	18	0	6	0	4	0	16	0	9
700	0	1400	0	900	0	8	0	8	0	8	0	0	12	0	16	0	4	0	0	0	0	0	0	0	1	0	13	0	1	0	5	0	18	0	6	0	4	0	16	0	9
700	0	1400	0	900	0	8	0	8	0	8	0	0	0	0	0	0	0	0	0	0	0	0	0	0	0	0	0	0	0	0	0	0	0	0	0	0	0	0	0	0	0
700	0	1400	0	900	0	8	0	8	0	8	0	0	4	0	4	0	1	0	0	0	0	0	0	0	0	0	5	0	0	0	1	0	11	0	1	0	1	0	8	0	4
840	0	1680	0	1080	0	9	0	9	0	9	0	0	4	0	4	0	1	0	0	0	0	0	0	0	0	0	5	0	0	0	1	0	11	0	1	0	1	0	8	0	4
840	0	1680	0	1080	0	9	0	9	0	9	0	0	0	0	0	0	0	0	0	0	0	0	0	0	0	0	0	0	0	0	0	0	0	0	0	0	0	0	0	0	0
840	0	1680	0	1080	0	9	0	9	0	9	0	0	1	0	1	0	0	0	0	0	0	0	0	0	0	0	1	0	0	0	0	0	3	0	0	0	0	0	3	0	1
980	0	1960	0	1260	0	10	0	10	0	10	0	0	1	0	1	0	0	0	0	0	0	0	0	0	0	0	1	0	0	0	0	0	3	0	0	0	0	0	3	0	1
980	0	1960	0	1260	0	10	0	10	0	10	0	0	0	0	0	0	0	0	0	0	0	0	0	0	0	0	0	0	0	0	0	0	0	0	0	0	0	0	0	0	0
980	0	1960	0	1260	0	10	0	10	0	10	0	0	0	0	0	0	0	0	0	0	0	0	0	0	0	0	1	0	0	0	0	0	1	0	0	0	0	0	1	0	1
1120	0	2240	0	1440	0	11	0	11	0	11	0	0	0	0	0	0	0	0	0	0	0	0	0	0	0	0	1	0	0	0	0	0	1	0	0	0	0	0	1	0	1
1120	0	2240	0	1440	0	11	0	11	0	11	0	0	0	0	0	0	0	0	0	0	0	0	0	0	0	0	0	0	0	0	0	0	0	0	0	0	0	0	0	0	0
1120	0	2240	0	1440	0	11	0	11	0	11	0	0	0	0	0	0	0	0	0	0	0	0	0	0	0	0	0	0	0	0	0	0	0	0	0	0	0	0	0	0	0
1260	0	2520	0	1620	0	11	0	12	0	12	0	0	0	0	0	0	0	0	0	0	0	0	0	0	0	0	0	0	0	0	0	0	0	0	0	0	0	0	0	0	0
1260	0	2520	0	1620	0	11	0	12	0	12	0	0	0	0	0	0	0	0	0	0	0	0	0	0	0	0	0	0	0	0	0	0	0	0	0	0	0	0	0	0	0
1260	0	2520	0	1620	0	11	0	12	0	12	0	0	0	0	0	0	0	0	0	0	0	0	0	0	0	0	0	0	0	0	0	0	0	0	0	0	0	0	0	0	0
1400	0	2800	0	1800	0	12	0	13	0	13	0	0	0	0	0	0	0	0	0	0	0	0	0	0	0	0	0	0	0	0	0	0	0	0	0	0	0	0	0	0	0
1400	0	2800	0	1800	0	12	0	13	0	13	0	0	0	0	0	0	0	0	0	0	0	0	0	0	0	0	0	0	0	0	0	0	0	0	0	0	0	0	0	0	0

0	0	0	0	0	0	0	0	0	0	0	0	0	0	0	0	0	0	0	0	0	0	0	0	0	0	0	0	0	0
0	0	0	0	0	0	0	1	0	2	0	3	0	0	0	0	0	0	0	0	0	0	0	1	0	1	0	0	0	1
0	0	0	0	0	0	0	1	0	2	0	3	0	0	0	0	0	0	2	0	1	0	2	1	0	1	0	0	0	1
0	0	0	0	0	0	0	0	0	0	0	0	0	0	0	0	0	0	2	0	1	0	2	0	0	0	0	0	0	0
0	3	0	1	0	3	0	8	0	17	0	9	0	3	0	2	0	3	2	0	1	1	2	0	0	3	0	2	0	3
0	3	0	1	0	3	0	8	0	17	0	9	0	3	0	2	0	3	5	0	1	1	3	0	0	3	0	2	0	3
0	0	0	0	0	0	0	0	0	0	0	0	0	0	0	0	0	0	5	0	1	0	3	0	0	0	0	0	0	0
0	13	0	7	0	11	0	18	0	13	0	22	0	11	0	6	0	12	5	0	1	0	3	0	0	2	0	2	0	10
0	13	0	7	0	11	0	18	0	13	0	22	0	11	0	6	0	12	7	0	2	0	5	0	0	2	0	2	0	10
0	0	0	0	0	0	0	0	0	0	0	0	0	0	0	0	0	0	7	0	2	0	5	0	0	0	0	0	0	0
0	13	0	17	0	10	0	9	0	2	0	23	0	12	0	15	0	16	7	0	2	0	5	0	0	5	0	3	0	3
0	13	0	17	0	10	0	9	0	2	0	23	0	12	0	15	0	16	9	0	2	0	7	0	0	5	0	3	0	3
0	0	0	0	0	0	0	0	0	0	0	0	0	0	0	0	0	0	9	0	2	0	7	0	0	0	0	0	0	0
0	5	0	10	0	4	0	2	0	1	0	10	0	6	0	18	0	6	9	0	2	0	7	0	0	9	0	12	0	0
0	5	0	10	0	4	0	2	0	1	0	10	0	6	0	18	0	6	12	0	3	0	9	0	0	9	0	12	0	0
0	0	0	0	0	0	0	0	0	0	0	0	0	0	0	0	0	0	12	0	3	0	9	0	0	0	0	0	0	0
0	2	0	4	0	1	0	1	0	0	0	4	0	2	0	11	0	2	12	0	3	0	9	0	0	4	0	14	0	0
0	2	0	4	0	1	0	1	0	0	0	4	0	2	0	11	0	2	14	0	4	0	10	0	0	4	0	14	0	0
0	0	0	0	0	0	0	0	0	0	0	0	0	0	0	0	0	0	14	0	4	0	10	0	0	0	0	0	0	0
0	0	0	2	0	0	0	0	0	0	0	1	0	0	0	4	0	0	14	0	4	0	10	0	0	1	0	5	0	0
0	0	0	2	0	0	0	0	0	0	0	1	0	0	0	4	0	0	16	0	4	0	12	0	0	1	0	5	0	0
0	0	0	0	0	0	0	0	0	0	0	0	0	0	0	0	0	0	16	0	4	0	12	0	0	0	0	0	0	0
0	0	0	0	0	0	0	0	0	0	0	0	0	0	0	1	0	0	16	0	4	0	12	0	0	0	0	1	0	0
0	0	0	0	0	0	0	0	0	0	0	0	0	0	0	1	0	0	18	0	5	0	14	0	0	0	0	1	0	0
0	0	0	0	0	0	0	0	0	0	0	0	0	0	0	0	0	0	18	0	5	0	14	0	0	0	0	0	0	0
0	0	0	0	0	0	0	0	0	0	0	0	0	0	0	0	0	0	18	0	5	0	14	0	0	0	0	0	0	0
0	0	0	0	0	0	0	0	0	0	0	0	0	0	0	0	0	0	21	0	5	0	15	0	0	0	0	0	1	0
0	0	0	0	0	0	0	0	0	0	0	0	0	0	0	0	0	0	21	0	5	0	15	0	0	0	0	0	1	0
0	0	0	0	0	0	0	0	0	0	0	0	0	0	0	0	0	0	21	0	5	0	15	0	0	0	0	0	1	0
0	0	0	0	0	0	0	0	0	0	0	0	0	0	0	0	0	0	23	0	6	0	17	0	0	0	0	0	1	0
0	0	0	0	0	0	0	0	0	0	0	0	0	0	0	0	0	0	23	0	6	0	17	0	0	0	0	0	1	0

-2	0	-2	0	-2	0	-1	0	-1	0	-1	0	1	0	1	0	1	0	60	0
-2	0	-2	0	-2	0	-1	0	-1	0	-1	0	1	0	1	0	1	0	60	0
-2	0	-2	0	-2	0	-1	0	-1	0	-1	0	1	0	1	0	1	0	63	0
-2	0	-2	0	-2	0	-1	0	-1	0	-1	0	1	0	1	0	1	0	63	0
-2	0	-2	0	-2	0	-1	0	-1	0	-1	0	1	0	1	0	1	0	63	0
-1	0	-1	0	-1	0	-1	0	-1	0	-1	0	1	0	1	0	1	0	66	0
-1	0	-1	0	-1	0	-1	0	-1	0	-1	0	1	0	1	0	1	0	66	0
-1	0	-1	0	-1	0	-1	0	-1	0	-1	0	1	0	1	0	1	0	66	0
-1	0	-1	0	-1	0	-1	0	-1	0	-1	0	1	0	1	0	1	0	69	0
-1	0	-1	0	-1	0	-1	0	-1	0	-1	0	1	0	1	0	1	0	69	0
-1	0	-1	0	-1	0	-1	1	-1	1	-1	0	1	3	1	3	1	3	69	0
-1	0	-1	0	-1	0	-1	1	-1	1	-1	0	1	3	1	3	1	3	73	0
-1	0	-1	0	-1	0	-1	0	-1	0	-1	0	1	0	1	0	1	0	73	0
-1	0	-1	0	-1	0	-1	5	-1	5	-1	4	1	7	1	9	1	10	73	0
-0	0	-0	0	-0	0	-1	5	-1	5	-1	4	1	7	1	9	1	10	76	0
-0	0	-0	0	-0	0	-1	0	-1	0	-1	0	1	0	1	0	1	0	76	0
-0	1	-0	0	-0	0	-1	7	-1	10	-1	5	1	8	1	6	1	7	76	0
0	1	-0	0	-0	0	-1	7	-1	10	-1	5	1	8	1	6	1	7	79	0
0	0	-0	0	-0	0	-1	0	-1	0	-1	0	1	0	1	0	1	0	79	0
0	1	-0	2	-0	2	-1	22	-1	18	-1	25	1	1	1	1	1	1	79	0
0	1	0	2	0	2	-1	22	-1	18	-1	25	1	1	1	1	1	1	82	0
0	0	0	0	0	0	-1	0	-1	0	-1	0	1	0	1	0	1	0	82	0
0	0	0	0	0	0	-1	2	-1	1	-1	6	1	0	1	0	1	0	82	0
1	0	1	0	1	0	-0	2	-0	1	-1	6	1	0	1	0	1	0	85	0
1	0	1	0	1	0	-0	0	-0	0	-1	0	1	0	1	0	1	0	85	0
1	0	1	0	1	0	-0	0	-0	0	-1	1	1	0	1	0	1	0	85	0
1	0	1	0	1	0	-0	0	-0	0	-0	1	1	0	1	0	1	0	88	0
1	0	1	0	1	0	-0	0	-0	0	-0	0	1	0	1	0	1	0	88	0
1	0	1	0	1	0	-0	0	-0	0	-0	0	1	0	1	0	1	0	88	0
1	0	1	0	1	0	-0	0	-0	0	-0	0	1	0	1	0	1	0	92	0
1	0	1	0	1	0	-0	0	-0	0	-0	0	1	0	1	0	1	0	92	0

60	0	60	0	1	0	1	0	1	0	-3	0	-3	0	-3	0	0	0	0	0
60	0	60	0	1	0	1	0	1	0	-3	0	-3	0	-3	0	0	0	0	0
63	0	63	0	1	0	1	0	1	0	-3	0	-2	0	-3	0	0	0	0	0
63	0	63	0	1	0	1	0	1	0	-3	0	-2	0	-3	0	0	0	0	0
63	0	63	0	1	0	1	0	1	0	-3	0	-2	0	-3	0	0	0	0	0
67	0	66	0	1	0	1	0	1	0	-2	0	-2	0	-2	0	0	0	0	0
67	0	66	0	1	0	1	0	1	0	-2	0	-2	0	-2	0	0	0	0	0
67	0	66	0	1	1	1	2	1	1	-2	0	-2	0	-2	0	0	0	0	0
70	0	69	0	1	1	1	2	1	1	-2	0	-1	0	-2	0	0	0	0	0
70	0	69	0	1	0	1	0	1	0	-2	0	-1	0	-2	0	0	0	0	0
70	0	69	0	1	6	1	9	1	4	-2	1	-1	1	-2	0	0	2	0	3
73	0	73	0	1	6	1	9	1	4	-1	1	-1	1	-1	0	0	2	0	3
73	0	73	0	1	0	1	0	1	0	-1	0	-1	0	-1	0	0	0	0	0
73	0	73	0	1	13	1	12	1	11	-1	1	-1	1	-1	1	0	14	0	30
77	0	76	0	1	13	1	12	1	11	-1	1	-0	1	-1	1	0	14	0	30
77	0	76	0	1	0	1	0	1	0	-1	0	-0	0	-1	0	0	0	0	0
77	0	76	0	1	4	1	2	1	12	-1	0	-0	0	-1	1	0	55	0	49
80	0	79	0	1	4	1	2	1	12	-0	0	0	0	-1	1	0	55	0	49
80	0	79	0	1	0	1	0	1	0	-0	0	0	0	-1	0	0	0	0	0
80	0	79	0	1	0	1	0	1	2	-0	0	0	0	-1	0	0	26	0	17
83	0	82	0	1	0	1	0	1	2	0	0	1	0	-0	0	0	26	0	17
83	0	82	0	1	0	1	0	1	0	0	0	1	0	-0	0	0	0	0	0
83	0	82	0	1	0	1	0	1	0	0	0	1	0	-0	0	0	13	0	6
87	0	85	0	1	0	1	0	1	0	1	0	1	0	0	0	0	13	0	6
87	0	85	0	1	0	1	0	1	0	1	0	1	0	0	0	0	0	0	0
87	0	85	0	1	0	1	0	1	0	1	0	1	0	0	0	0	5	0	1
90	0	88	0	1	0	1	0	1	0	1	0	2	0	1	0	0	5	0	1
90	0	88	0	1	0	1	0	1	0	1	0	2	0	1	0	0	0	0	0
90	0	88	0	1	0	1	0	1	0	1	0	2	0	1	0	0	1	0	0
93	0	91	0	1	0	1	0	1	0	2	0	2	0	1	0	0	1	0	0
93	0	91	0	1	0	1	0	1	0	2	0	2	0	1	0	0	0	0	0

0	0	70	0	70	0	70	0	-0	0	-0	0	-0	0	0	0	0	0	0	0
0	0	70	0	70	0	70	0	-0	0	-0	0	-0	0	0	0	0	0	0	0
0	0	73	0	73	0	73	0	-0	0	-0	0	-0	0	240	0	550	0	160	0
0	0	73	0	73	0	73	0	-0	0	-0	0	-0	0	240	0	550	0	160	0
0	0	73	0	73	0	73	0	-0	0	-0	0	-0	0	240	0	550	0	160	0
0	0	75	0	75	0	75	0	-0	0	-0	0	-0	0	480	0	1100	0	320	0
0	0	75	0	75	0	75	0	-0	0	-0	0	-0	0	480	0	1100	0	320	0
0	0	75	0	75	0	75	0	-0	0	-0	0	-0	0	480	0	1100	0	320	0
0	0	78	0	78	0	78	0	-0	0	-0	0	-0	0	720	0	1650	0	480	0
0	0	78	0	78	0	78	0	-0	0	-0	0	-0	0	720	0	1650	0	480	0
0	2	78	0	78	0	78	0	-0	1	-0	3	-0	0	720	0	1650	0	480	0
0	2	80	0	80	0	80	0	-0	1	-0	3	-0	0	960	0	2200	0	640	0
0	0	80	0	80	0	80	0	-0	0	-0	0	-0	0	960	0	2200	0	640	0
0	11	80	0	80	0	80	0	-0	7	-0	9	-0	8	960	0	2200	0	640	0
0	11	83	0	83	0	83	0	0	7	0	9	0	8	1200	0	2750	0	800	0
0	0	83	0	83	0	83	0	0	0	0	0	0	0	1200	0	2750	0	800	0
0	50	83	0	83	0	83	0	0	3	0	1	0	3	1200	0	2750	0	800	0
0	50	86	0	86	0	85	0	0	3	0	1	0	3	1440	0	3300	0	960	0
0	0	86	0	86	0	85	0	0	0	0	0	0	0	1440	0	3300	0	960	0
0	35	86	0	86	0	85	0	0	0	0	0	0	0	1440	0	3300	0	960	0
0	35	88	0	88	0	88	0	0	0	0	0	0	0	1680	0	3850	0	1120	0
0	0	88	0	88	0	88	0	0	0	0	0	0	0	1680	0	3850	0	1120	0
0	11	88	0	88	0	88	0	0	0	0	0	0	0	1680	0	3850	0	1120	0
0	11	91	0	91	0	90	0	0	0	0	0	0	0	1920	0	4400	0	1280	0
0	0	91	0	91	0	90	0	0	0	0	0	0	0	1920	0	4400	0	1280	0
0	1	91	0	91	0	90	0	0	0	0	0	0	0	1920	0	4400	0	1280	0
0	1	94	0	93	0	93	0	0	0	0	0	0	0	2160	0	4950	0	1440	0
0	0	94	0	93	0	93	0	0	0	0	0	0	0	2160	0	4950	0	1440	0
0	0	94	0	93	0	93	0	0	0	0	0	0	0	2160	0	4950	0	1440	0
0	0	96	0	96	0	95	0	0	0	0	0	0	0	2400	0	5500	0	1600	0
0	0	96	0	96	0	95	0	0	0	0	0	0	0	2400	0	5500	0	1600	0

3	0	3	0	3	0	0	0	0	0	0	0	0	0	0	0	0	0	0	0
3	0	3	0	3	0	0	5	0	5	0	6	0	11	0	12	0	9	0	3
4	0	4	0	4	0	0	5	0	5	0	6	0	11	0	12	0	9	0	3
4	0	4	0	4	0	0	0	0	0	0	0	0	0	0	0	0	0	0	0
4	0	4	0	4	0	0	9	0	11	0	15	0	18	0	16	0	23	0	5
5	0	5	0	5	0	0	9	0	11	0	15	0	18	0	16	0	23	0	5
5	0	5	0	5	0	0	0	0	0	0	0	0	0	0	0	0	0	0	0
5	0	5	0	5	0	0	19	0	19	0	17	0	3	0	2	0	7	0	14
6	0	6	0	6	0	0	19	0	19	0	17	0	3	0	2	0	7	0	14
6	0	6	0	6	0	0	0	0	0	0	0	0	0	0	0	0	0	0	0
6	0	6	0	6	0	0	11	0	7	0	4	0	0	0	0	0	1	0	9
7	0	7	0	7	0	0	11	0	7	0	4	0	0	0	0	0	1	0	9
7	0	7	0	7	0	0	0	0	0	0	0	0	0	0	0	0	0	0	0
7	0	7	0	7	0	0	2	0	1	0	1	0	0	0	0	0	0	0	2
8	0	8	0	8	0	0	2	0	1	0	1	0	0	0	0	0	0	0	2
8	0	8	0	8	0	0	0	0	0	0	0	0	0	0	0	0	0	0	0
8	0	8	0	8	0	0	0	0	0	0	0	0	0	0	0	0	0	0	1
9	0	9	0	9	0	0	0	0	0	0	0	0	0	0	0	0	0	0	1
9	0	9	0	9	0	0	0	0	0	0	0	0	0	0	0	0	0	0	0
9	0	9	0	9	0	0	0	0	0	0	0	0	0	0	0	0	0	0	0
10	0	10	0	10	0	0	0	0	0	0	0	0	0	0	0	0	0	0	0
10	0	10	0	10	0	0	0	0	0	0	0	0	0	0	0	0	0	0	0
10	0	10	0	10	0	0	0	0	0	0	0	0	0	0	0	0	0	0	0
11	0	11	0	11	0	0	0	0	0	0	0	0	0	0	0	0	0	0	0
11	0	11	0	11	0	0	0	0	0	0	0	0	0	0	0	0	0	0	0
11	0	11	0	11	0	0	0	0	0	0	0	0	0	0	0	0	0	0	0
12	0	12	0	12	0	0	0	0	0	0	0	0	0	0	0	0	0	0	0
12	0	12	0	12	0	0	0	0	0	0	0	0	0	0	0	0	0	0	0
12	0	12	0	12	0	0	0	0	0	0	0	0	0	0	0	0	0	0	0
13	0	13	0	13	0	0	0	0	0	0	0	0	0	0	0	0	0	0	0
13	0	13	0	13	0	0	0	0	0	0	0	0	0	0	0	0	0	0	0

0	0	0	0	0	0	0	0	0	0	0	0	0	0	0	0	0	0	0	0
0	3	0	3	0	22	0	24	0	18	0	0	0	0	0	0	0	1	0	1
0	3	0	3	0	22	0	24	0	18	0	0	0	0	0	0	0	1	0	1
0	0	0	0	0	0	0	0	0	0	0	0	0	0	0	0	0	0	0	0
0	6	0	5	0	30	0	16	0	30	0	1	0	1	0	2	0	4	0	5
0	6	0	5	0	30	0	16	0	30	0	1	0	1	0	2	0	4	0	5
0	0	0	0	0	0	0	0	0	0	0	0	0	0	0	0	0	0	0	0
0	10	0	12	0	17	0	2	0	23	0	6	0	7	0	10	0	14	0	16
0	10	0	12	0	17	0	2	0	23	0	6	0	7	0	10	0	14	0	16
0	0	0	0	0	0	0	0	0	0	0	0	0	0	0	0	0	0	0	0
0	14	0	14	0	5	0	0	0	9	0	16	0	14	0	13	0	25	0	24
0	14	0	14	0	5	0	0	0	9	0	16	0	14	0	13	0	25	0	24
0	0	0	0	0	0	0	0	0	0	0	0	0	0	0	0	0	0	0	0
0	5	0	5	0	1	0	0	0	3	0	11	0	11	0	7	0	15	0	13
0	5	0	5	0	1	0	0	0	3	0	11	0	11	0	7	0	15	0	13
0	0	0	0	0	0	0	0	0	0	0	0	0	0	0	0	0	0	0	0
0	2	0	1	0	0	0	0	0	1	0	5	0	5	0	2	0	4	0	4
0	2	0	1	0	0	0	0	0	1	0	5	0	5	0	2	0	4	0	4
0	0	0	0	0	0	0	0	0	0	0	0	0	0	0	0	0	0	0	0
0	1	0	0	0	0	0	0	0	0	0	2	0	2	0	0	0	1	0	1
0	1	0	0	0	0	0	0	0	0	0	2	0	2	0	0	0	1	0	1
0	0	0	0	0	0	0	0	0	0	0	0	0	0	0	0	0	0	0	0
0	0	0	0	0	0	0	0	0	0	0	0	0	0	0	0	0	0	0	0
0	0	0	0	0	0	0	0	0	0	0	0	0	0	0	0	0	0	0	0
0	0	0	0	0	0	0	0	0	0	0	0	0	0	0	0	0	0	0	0
0	0	0	0	0	0	0	0	0	0	0	0	0	0	0	0	0	0	0	0
0	0	0	0	0	0	0	0	0	0	0	0	0	0	0	0	0	0	0	0
0	0	0	0	0	0	0	0	0	0	0	0	0	0	0	0	0	0	0	0
0	0	0	0	0	0	0	0	0	0	0	0	0	0	0	0	0	0	0	0
0	0	0	0	0	0	0	0	0	0	0	0	0	0	0	0	0	0	0	0
0	0	0	0	0	0	0	0	0	0	0	0	0	0	0	0	0	0	0	0

0	0	0	0	0	0	0	0	0	0	0	0	0	0	0	0	0	0	0	0
0	2	0	1	0	1	0	1	0	1	0	0	0	0	0	1	0	1	0	1
0	2	0	1	0	1	0	1	1	1	1	0	1	0	0	1	0	1	0	1
0	0	0	0	0	0	0	0	1	0	1	0	1	0	0	0	0	0	0	0
0	9	0	3	0	4	0	3	1	0	1	1	1	1	0	4	0	3	0	3
0	9	0	3	0	4	0	3	2	0	2	1	1	1	0	4	0	3	0	3
0	0	0	0	0	0	0	0	2	0	2	0	1	0	0	0	0	0	0	0
0	24	0	12	0	12	0	10	2	0	2	0	1	0	0	3	0	4	0	6
0	24	0	12	0	12	0	10	4	0	2	0	2	0	0	3	0	4	0	6
0	0	0	0	0	0	0	0	4	0	2	0	2	0	0	0	0	0	0	0
0	16	0	18	0	18	0	17	4	0	2	0	2	0	0	10	0	12	0	12
0	16	0	18	0	18	0	17	5	0	3	0	2	0	0	10	0	12	0	12
0	0	0	0	0	0	0	0	5	0	3	0	2	0	0	0	0	0	0	0
0	4	0	13	0	12	0	12	5	0	3	0	2	0	0	7	0	4	0	3
0	4	0	13	0	12	0	12	6	0	4	0	3	0	0	7	0	4	0	3
0	0	0	0	0	0	0	0	6	0	4	0	3	0	0	0	0	0	0	0
0	1	0	5	0	4	0	4	6	0	4	0	3	0	0	2	0	1	0	1
0	1	0	5	0	4	0	4	7	0	5	0	4	0	0	2	0	1	0	1
0	0	0	0	0	0	0	0	7	0	5	0	4	0	0	0	0	0	0	0
0	0	0	1	0	1	0	1	7	0	5	0	4	0	0	0	0	0	0	0
0	0	0	1	0	1	0	1	8	0	5	0	4	0	0	0	0	0	0	0
0	0	0	0	0	0	0	0	8	0	5	0	4	0	0	0	0	0	0	0
0	0	0	0	0	0	0	0	8	0	5	0	4	0	0	0	0	0	0	0
0	0	0	0	0	0	0	0	10	0	6	0	5	0	0	0	0	0	0	0
0	0	0	0	0	0	0	0	10	0	6	0	5	0	0	0	0	0	0	0
0	0	0	0	0	0	0	0	10	0	6	0	5	0	0	0	0	0	0	0
0	0	0	0	0	0	0	0	11	0	7	0	5	0	0	0	0	0	0	0
0	0	0	0	0	0	0	0	11	0	7	0	5	0	0	0	0	0	0	0
0	0	0	0	0	0	0	0	11	0	7	0	5	0	0	0	0	0	0	0
0	0	0	0	0	0	0	0	12	0	8	0	6	0	0	0	0	0	0	0
0	0	0	0	0	0	0	0	12	0	8	0	6	0	0	0	0	0	0	0
